# Phosphorylation of Elp1 by Hrr25 Is Required for Elongator-Dependent tRNA Modification in Yeast

**DOI:** 10.1371/journal.pgen.1004931

**Published:** 2015-01-08

**Authors:** Wael Abdel-Fattah, Daniel Jablonowski, Rachael Di Santo, Kathrin L. Thüring, Viktor Scheidt, Alexander Hammermeister, Sara ten Have, Mark Helm, Raffael Schaffrath, Michael J. R. Stark

**Affiliations:** 1 Centre for Gene Regulation & Expression, College of Life Sciences, University of Dundee, Dundee, United Kingdom; 2 Institut für Biologie, FG Mikrobiologie, Universität Kassel, Germany; 3 Institut für Pharmazie und Biochemie, Johannes Gutenberg-Universität Mainz, Germany; 4 Department of Genetics, University of Leicester, Leicester, United Kingdom; University of Kent, United Kingdom

## Abstract

Elongator is a conserved protein complex comprising six different polypeptides that has been ascribed a wide range of functions, but which is now known to be required for modification of uridine residues in the wobble position of a subset of tRNAs in yeast, plants, worms and mammals. In previous work, we showed that Elongator's largest subunit (Elp1; also known as Iki3) was phosphorylated and implicated the yeast casein kinase I Hrr25 in Elongator function. Here we report identification of nine *in vivo* phosphorylation sites within Elp1 and show that four of these, clustered close to the Elp1 C-terminus and adjacent to a region that binds tRNA, are important for Elongator's tRNA modification function. Hrr25 protein kinase directly modifies Elp1 on two sites (Ser-1198 and Ser-1202) and through analyzing non-phosphorylatable (alanine) and acidic, phosphomimic substitutions at Ser-1198, Ser-1202 and Ser-1209, we provide evidence that phosphorylation plays a positive role in the tRNA modification function of Elongator and may regulate the interaction of Elongator both with its accessory protein Kti12 and with Hrr25 kinase.

## Author Summary

tRNA molecules function as adapters in protein synthesis, bringing amino acids to the ribosome and reading the genetic code through codon-anticodon base pairing. When the tRNA contains a uridine residue in the “wobble position” of its anticodon, which base-pairs with purine residues in the third position of a cognate codon, it is almost always chemically modified and modification is required for efficient decoding. In eukaryotic cells, these wobble uridine modifications require a conserved protein complex called Elongator. Our work shows that Elp1, Elongator's largest subunit, is phosphorylated on several sites. By blocking phosphorylation at these positions using mutations, we identified four phosphorylation sites that are important for Elongator's role in tRNA modification. We have also shown that Hrr25 protein kinase, a member of the casein kinase I (CKI) family, is responsible for modification of two of the sites that are important for Elongator function. Phosphorylation appears to affect interaction of the Elongator complex both with its kinase (Hrr25) and with Kti12, an accessory protein previously implicated in Elongator function. Our studies imply that Elp1 phosphorylation plays a positive role in Elongator-mediated tRNA modification and raise the possibility that wobble uridine modification may be regulated, representing a potential translational control mechanism.

## Introduction

Elongator is a conserved, multi-subunit protein complex containing six different polypeptides (Elp1-Elp6), first discovered in yeast in association with the elongating form of RNA polymerase II and initially proposed to play a role in transcriptional elongation [1], [2]. Although Elongator is non-essential in yeast, knockout of the mouse *IKBKAP* gene encoding Elongator's largest subunit leads to embryonic lethality and the protein is crucial for vascular and neural development [3]. The hereditary neuropathy Familial Dysautonomia results from human *IKBKAP* mutations, while mutations in other Elongator subunits have been associated with Amyotrophic Lateral Sclerosis [4] and Rolandic Epilepsy [5]. Elongator in *Caenorhabditis elegans* is also involved in neuronal function and development [6], [7], while in plants it plays a role in proliferation during organ growth [8].

While it is therefore clear that Elongator is important for neural function in higher organisms [9], [10], it has been proposed to have a bewildering range of seemingly unrelated functions. The Elp3 subunit of Elongator has a histone acetyltransferase (HAT) domain that can acetylate histones *in vitro* [11] and yeast Elongator mutants show changes in histone acetylation *in vivo* [12]. Elongator has also been proposed to acetylate α-tubulin and the neuronal protein Bruchpilot [7], [13], [14] and has been implicated in paternal DNA demethylation in mouse zygotes [15]. In yeast, Elongator mutants adapt slowly to changing growth conditions, show sensitivity to high temperature, rapamycin, caffeine, hydroxyurea and various other chemical stressors, and are resistant to zymocin [16], [17], a protein toxin secreted by the yeast *Kluyveromyces lactis* that kills other yeasts including *Saccharomyces cerevisiae* [18]. Yeast Elongator has also been implicated in transcriptional silencing, replication-coupled nucleosome assembly [17] and polarized secretion [19].

However, while these apparently diverse roles could imply that Elongator is multifunctional, work in yeast [20], *C. elegans* [6], plants [21] and mammals [22] has demonstrated that many if not all of these proposed functions reflect a primary role for Elongator in tRNA modification, specifically in the addition of mcm^5^ (5-methoxycarbonylmethyl) and ncm^5^ (5-carbamoylmethyl) groups to uridine when present at the ‘wobble’ position (U34) in tRNA anticodons. Eleven out of 13 such tRNAs in yeast contain either mcm^5^U, ncm^5^U or 5-methoxycarbonymethyl-2-thiouridine (mcm^5^s^2^U) in the wobble position and addition of the mcm^5^ and ncm^5^ moieties requires Elongator [20]. This role in tRNA modification explains the zymocin-resistant phenotype of Elongator mutants: zymocin is a tRNA anticodon nuclease that inactivates tRNA^Glu^(UUC) by cleaving the anticodon on the 3′ side of U34, but the mcm^5^ modification is necessary for tRNA recognition and cleavage [23], [24].

Wobble uridine-containing tRNAs read codons ending with a purine, and the mcm^5^/ncm^5^ modifications are needed to confer full decoding competence on these tRNAs [6], [20], [25]-[27]. Wobble uridine modification is most likely the primary role of Elongator, at least in yeast, because elevated expression of just two Elongator-dependent tRNAs, tRNA^Lys^(UUU) and tRNA^Gln^(UUG), can suppress all the phenotypes associated with loss of Elongator function apart from zymocin resistance, which remains unaffected because elevated levels of the two tRNAs do not restore the tRNA^Glu^(UUC) modification required for cleavage by zymocin [28], [29]. Suppression of Elongator mutant phenotypes by elevated tRNA levels without restoration of wobble uridine modification strongly suggests that these phenotypes are caused by translational defects resulting from hypomodified tRNAs, a notion supported by findings that U34 modification promotes binding of tRNA^Lys^(UUU), tRNA^Gln^(UUG) and tRNA^Glu^(UUC) to the ribosomal A-site [30]. Recent structural work indicates that Elp4, Elp5 and Elp6 are RecA-fold proteins that form a heterohexamer containing two copies of each polypeptide, which interacts with two copies of an Elp1-Elp2-Elp3 sub-complex [31]. The recombinant heterohexamer binds and hydrolyses NTPs and shows tRNA binding that is reduced when the NTP can be hydrolyzed [31], while a separate tRNA-binding motif in the C-terminal domain of Elp1 may also mediate tRNA interaction with the Elp1-Elp2-Elp3 sub-complex [32]. Thus while the existence of additional substrates cannot be excluded, it is now clear that Elongator plays a conserved role in wobble uridine modification [6], [21], [22] and that this role, through effects at the level of translation, is likely to underpin the majority of phenotypes resulting from Elongator deficiency, at least in yeast. Within Elongator, Elp3 is highly likely to catalyze the tRNA modification as it contains a ‘radical SAM’ domain [33] that in other proteins can mediate RNA modification reactions [34], and both its radical SAM and histone acetyltransferase (HAT) domains are required for wobble uridine modification [20], [29]. This is supported by the recent finding that recombinant archaeal Elp3 can catalyze modification of tRNA wobble uridines in an *in vitro* reaction containing SAM, acetyl-CoA, tRNA and Na_2_S_2_O_4_ [35].

Previously, we reported that the largest subunit of Elongator (Elp1) is a phosphoprotein and identified mutations in either *HRR25* (encoding a casein kinase I) or *SIT4* (encoding a protein phosphatase) that conferred zymocin resistance [36]-[38]. Elp1 was present as a hypophosphorylated isoform in *hrr25* mutants and as a hyperphosphorylated isoform in *sit4* mutants, whereas wild-type cells contained similar amounts of both isoforms [37], [39]. We therefore sought to investigate the potential functional significance of Elp1 phosphorylation by locating the phosphorylation sites on Elp1, identifying several such sites that are critical for Elongator-dependent tRNA modification. Our findings therefore raise the possibility that Elongator activity (and hence tRNA modification) could be regulated, potentially constituting a novel mechanism for translational control.

## Results

### Identification of *in vivo* phosphorylation sites in Elp1 and other Elongator subunits

To identify sites of phosphorylation in Elongator we affinity isolated the complex from yeast cells expressing a TAP-tagged version of Elp1. Tryptic digests of the affinity-purified material were subjected to phosphopeptide enrichment involving a two-step procedure using Hypersep SCX and TiO_2_, followed by tandem mass spectrometry to locate sites of phosphorylation. To maximize the chance of detecting phosphopeptides, in addition to isolating Elongator from wild-type yeast cells we also prepared and analyzed the complex from a *sit4Δ* strain, in which lack of Sit4 phosphatase leads to Elp1 hyperphosphorylation [37], [39]. We also analyzed Elongator prepared from a *kti12Δ* mutant in which Elongator is hypophosphorylated [37], [39] in case additional phosphorylation sites could also be detected under these circumstances. In this way we identified eight phosphorylation sites in Elp1, one each in Elp2 and Elp4 and two in Elp5 (Table 1). With the exception of Ser-222 in Elp4 [40], all of these sites are novel and were not identified in any of the recent proteome-wide phosphoproteomics studies.

**Table 1:** Summary of phosphorylation sites identified in Elp1 and other Elongator subunits.

Elp1		Elp2	Elp4	Elp5
Ser-529 (wt)^1^	Ser-1198 (wt, k)	Ser-492 (s)	Ser-222^4^ (s)	Ser-3 (w, s)
Ser-539 (wt)	Ser-1202 (wt)			Ser-4 (w, s)
Ser-551 (wt)	Ser-1205/Thr-1206^2^ (wt, s)			
Ser-636 (wt, k)	Ser-1209^3^			
Ser-828 (wt, s)				

1 For sites identified by mass spectrometry: wt, identified in Elongator from wild-type yeast cells; k, identified in Elongator from a *kti12Δ* strain; s, identified in Elongator from a *sit4Δ* mutant.

2 Phosphorylation on these two adjacent sites cannot be unambiguously distinguished.

3 Demonstrated using a phosphospecific antibody; all other sites identified by mass spectrometry.

4 Previously identified by Soulard *et al*.[40].

Since we previously showed that Elp1 phosphorylation state changes are associated with altered Elongator function [37], [39], we focused on the phosphorylation sites we identified in Elp1. S1 Fig. shows representative MS/MS spectra providing evidence for the eight sites identified in Elp1, which with one exception were identified within monophosphorylated peptides. Apart from Ser-1205/Thr-1206, where phosphorylation of the two sites cannot be unambiguously distinguished from the mass spectra, all phosphorylation sites can be identified with high confidence. All Elp1 sites identified in Elongator isolated from either the *sit4Δ* mutant or the *kti12Δ* mutant were also found in Elp1 from the wild-type strain (Table 1).

**Figure 1:**
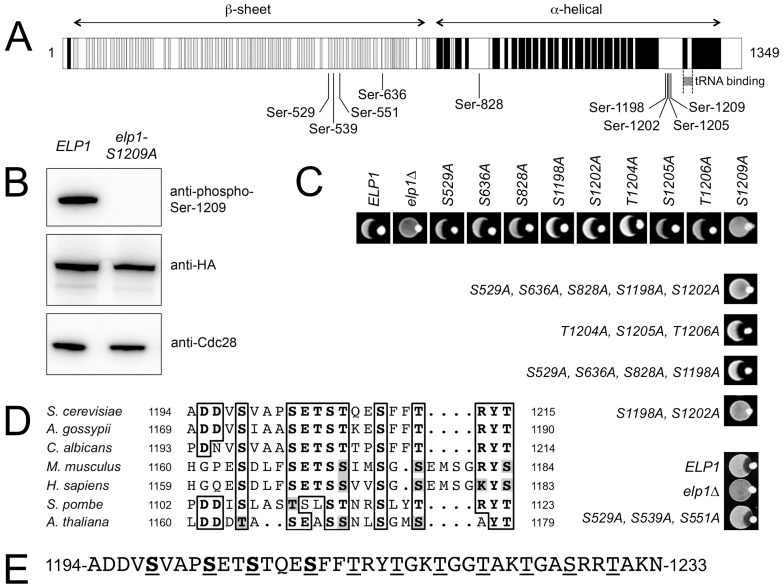
Identification of functionally important sites of phosphorylation in Elp1. (A) Cartoon showing Elp1 indicating mapped sites of phosphorylation and structural elements predicted using PSIPRED [82], with predicted α-helix shown in black and β-sheet in grey. (B) Western blot of cell lysate prepared from cells expressing *ELP1*-6HA (*ELP1*) or *elp1-S1209A*-6HA showing recognition of Elp1 by a phosphospecific antibody raised against the phosphopeptide TSTQE(pS)FFTRY that recognizes Elp1 phosphorylated on Ser-1209; blots with anti-HA and anti-Cdc28 antibodies control for levels of Elp1 and total protein loading respectively. (C) Eclipse assays showing the zymocin phenotype of wild-type and a range of single or multiple *elp1* phosphorylation site mutants. Lack of growth inhibition around the *K. lactis* colony placed at the edge of each patch of cells being tested indicates zymocin resistance and hence loss of Elongator function. (D) Conservation of the phosphorylation sites identified in the Elp1 C-terminal domain. The full sequences were first aligned using T-Coffee [83] but only the relevant detail from the alignment is shown. NCBI database accessions for the aligned sequences are *Saccharomyces cerevisiae* (NP_013488), *Ashbya gossypii* (NP_984907), *Candida albicans* (XP_710040), *Mus musculus* (NP_080355), *Homo sapiens* (NP_003631) *Schizosaccharomyces pombe* (NP_595335), *Arabidopsis thaliana* (NP_196872). (E) C-terminal phosphorylated region of *S. cerevisiae* Elp1 showing the downstream region with a repeating pattern of serine and threonine residues. Mapped phosphorylation sites are underlined and shown in bold, potential phosphorylation sites downstream of the mapped sites are underlined.

Since analysis of our mass spectrometry data also provided additional weak evidence for phosphorylation on Elp1 Ser-1209, we raised a phosphospecific antibody against a synthetic peptide carrying phosphate on this residue to examine whether it was a genuine phosphorylation site. As shown in Fig. 1B, when used to probe Elp1 by Western blotting, this phosphospecific antibody gave a strong signal that was lost when Ser-1209 was mutated to alanine, thereby demonstrating its phosphospecificity. Thus Elp1 Ser-1209 represents a ninth Elp1 phosphorylation site (Table 1). Fig. 1A shows that five of the nine sites are located centrally within Elp1. Four of these five sites map to the Elp1 amino-terminal domain, which is strongly predicted to form a β-propeller structure that may mediate interactions with other Elongator subunits or accessory proteins. The four remaining phosphorylation sites are tightly clustered close to the carboxy-terminus of Elp1, in a region that is located adjacent to its tRNA binding region [32] and predicted to be disordered (Fig. 1A). Phosphorylation sites are generally found to be enriched in disordered regions [41], in particular those sites that show dynamic variation in phosphorylation state [42]. Beyond Ser-1209, it is striking that every fourth residue between Thr-1212 and Thr-1230 is either threonine or serine (Fig. 1E). However, despite this intriguing pattern of phosphorylatable residues we were unable to detect phosphate groups in this region.

To determine whether any of the Elp1 phosphorylation sites we had identified were likely to be functionally significant, we mutated each in turn to non-phosphorylatable alanine. These mutants were assayed for zymocin sensitivity by eclipse assay, in which growth inhibition of an *S. cerevisiae* strain around a colony of zymocin-producing *K. lactis* indicates loss of Elongator-dependent mcm^5^ modification of tRNA^Glu^(UUC) [16], [24]. Fig. 1C shows that with the exception of the S1209A mutant, no alanine substitution at a single phosphorylation site conferred detectable resistance to zymocin and hence loss of Elongator function. In contrast, the S1209A substitution conferred complete zymocin resistance in this assay. Combinations of alanine substitutions at multiple sites were therefore also generated and tested (Fig. 1C and S1 Table). This analysis indicated that any mutants in which both Ser-1198 and Ser-1202 had been replaced by alanine were also zymocin-resistant. Thus Ser-1198, Ser-1202 and Ser-1209 define three phosphorylated residues in Elp1 that are required for Elongator function, but with Ser-1198 and Ser-1202 apparently showing some redundancy. Furthermore, these residues are highly conserved in Elp1 in both lower and higher eukaryotes (Fig. 1D). Since Elp1 becomes hyperphosphorylated in cells lacking Sit4 phosphatase that are defective for Elongator function [37], [39], we also examined whether any alanine substitutions could reverse the zymocin resistance shown by a *sit4Δ* mutant. However, none of the mutations tested altered the zymocin-resistance phenotype of the *sit4Δ* strain (S1 Table). Similarly, we also tried mimicking constitutive phosphorylation at many of the sites by making glutamate or aspartate substitutions alone or in combination (S1 Table), but failed to find any substitution(s) that conferred a significant loss of function phenotype.

### Phenotype of Elp1 C-terminal domain phosphorylation site mutants

To investigate the Elp1 C-terminal phosphorylated region, additional combinations of alanine, aspartate or glutamate substitutions at the phosphorylation sites were generated. In addition to using the eclipse assay, we monitored tolerance to intracellular expression of the zymocin tRNase (γ) subunit from the galactose-inducible *GAL1* promoter as a more quantitative measure of zymocin resistance [43]. Since zymocin sensitivity provides a readout largely for the mcm^5^ modification state of just tRNA^Glu^(UUC), we also monitored Elongator function via efficiency of *ochre* suppression mediated by *SUP4*, a tRNA^Tyr^(UUA) that requires Elongator-dependent wobble uridine modification for efficient *ochre* (UAA) codon readthrough [20], [24]. This involved single copy integration of a plasmid that carried both *SUP4* and a *ura3 ochre* allele [32], such that suppression (and hence Elongator's tRNA modification function) could be monitored by growth in the absence of uracil.

**Figure 2:**
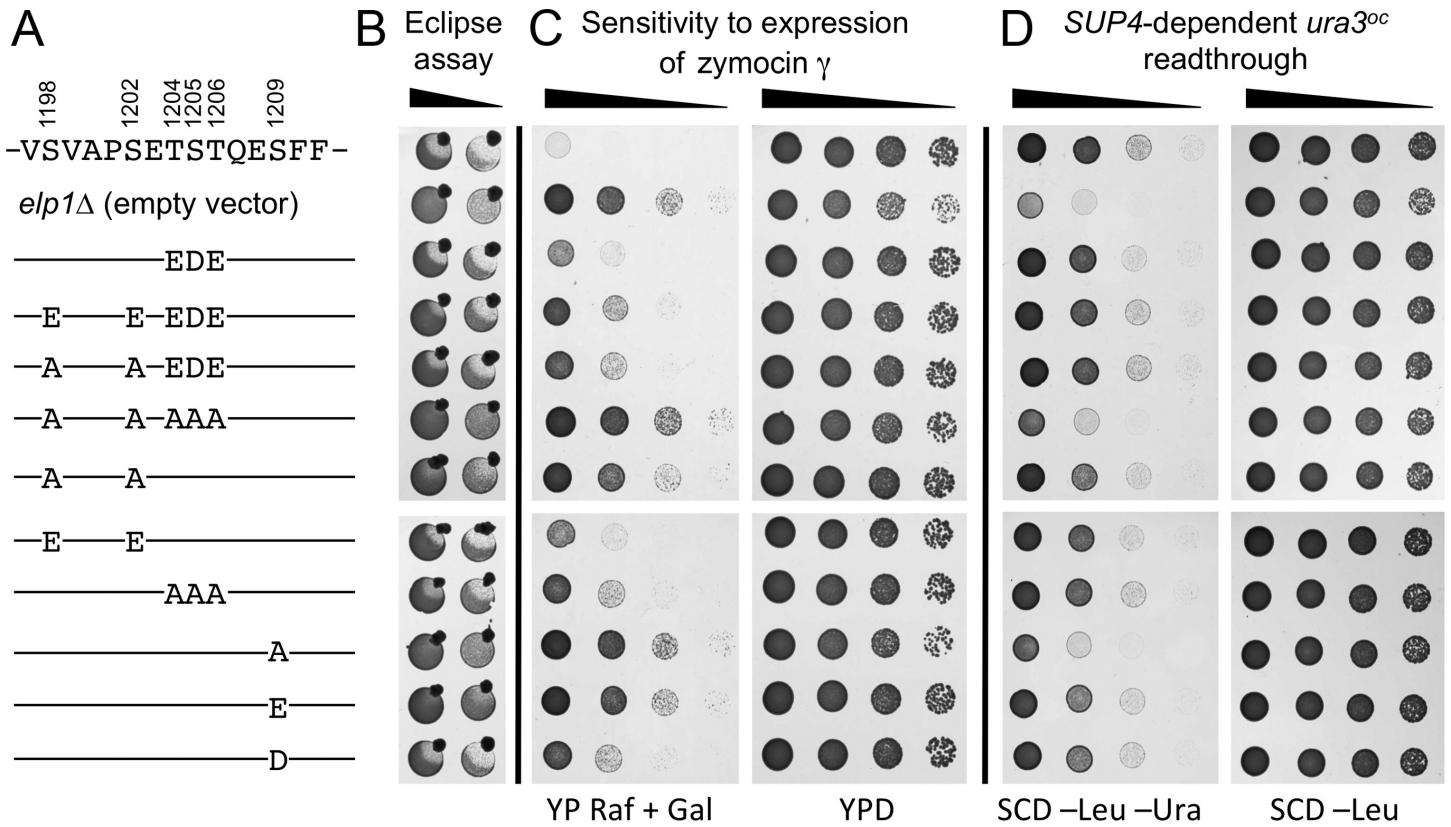
Effect on Elongator function of phosphorylation site mutations in the C-terminal domain of Elp1. All strains were based on WAY034 (*elp1Δ*; panel B), YRDS119 (*elp1Δ* [pAE1]; panel C) or SBY138 (*elp1Δ his3Δ1::*pSB3; panel D) transformed with YCplac111, YCplac111-*ELP1*-6HA (wild-type) or mutant derivatives carrying mutations in *ELP1* as shown. (A) Key to substitutions present in the different alleles tested (wild-type sequence at the top). (B) Zymocin sensitivity measured by eclipse assay (see Fig. 1 legend), with strains being tested at either 1.0 or 0.1 OD_600_/ml following growth in SCD-Leu medium. (C) Zymocin sensitivity monitored by intracellular expression of the zymocin γ subunit. Cells were grown on SCD-Leu-Ura to select for both plasmids, diluted to 1.0 OD_600_/ml and then equivalent 10-fold dilutions plated on YPD agar (to control for cell viability) and YP agar containing 2% raffinose and 2% galactose to induce expression of zymocin γ-toxin from pAE1. (D) Cells were grown in SCD-Leu (to select for YCplac111 and its derivatives), diluted to 1.0 OD_600_/ml and then equivalent 10-fold dilutions plated on SCD-Leu (to control for cell viability) and SCD-Leu-Ura (to assess efficiency of *SUP4*-dependent *ura3^oc22^* suppression.

Fig. 2 shows that the S1209A substitution conferred complete resistance to intracellular expression of the zymocin γ subunit and greatly reduced *SUP4*-dependent *ura3^oc^* suppression, in each case conferring a phenotype comparable to that observed upon complete loss of Elongator function (*elp1Δ*). In contrast, an S1209D substitution showed considerable restoration of function, while the equivalent glutamate substitution (potentially a poorer mimic of phosphoserine due to its longer side chain) was less effective. The regain of functionality caused by the phosphomimic aspartate substitution therefore provides evidence that phosphorylation of Ser-1209 acts positively for Elongator function. Although the double S1198A S1202A substitution mutant conferred strong zymocin resistance it showed considerable residual Elongator function in the *SUP4* assay, allowing for growth in the absence of uracil similar to that shown by the *ELP1* wild-type control. However, concurrent glutamate substitutions at both positions largely restored zymocin sensitivity, supporting the notion that phosphorylation of these two sites acts positively for Elongator function. S1198A S1202A in combination with T1204A S1205A T1206A, a triple alanine substitution that on its own had essentially no effect on Elongator function in any of the assays, conferred stronger zymocin resistance than S1198A S1202A alone and dramatically reduced *SUP4*-dependent suppression efficiency, indicative of an additive Elongator defect in the quintuple mutant. The triple T1204A S1205A T1206A mutation was used here because phosphorylation at Ser-1205/Thr-1206 could not be unambiguously distinguished (see above). Mutants where acidic residues substituted combinations of these five positions, either alone or in combination with alanine substitutions, indicated that Elongator functionality was not greatly affected by acidic substitutions, although the T1204E S1205D T1206E triple substitution improved Elongator function when combined with the S1198A S1202A double mutant (seen most clearly by the eclipse assay). While the difference in the severity of phenotype observed between the zymocin- and *SUP4*-based assays for some *elp1* mutants might reflect differential effects on tRNA^Glu^(UUC) and tRNA^Tyr^(UUA), we consider it more likely to reflect different loss of modification thresholds required to score positive in these assays; while as little as ∼40% reduction in modification may generate sufficient uncleavable tRNA^Glu^(UUC) to confer zymocin resistance [44], a much larger reduction in modification may be required before there is insufficient functional tRNA^Tyr^(UUA) to support effective *ochre* suppression. Our results therefore indicate that blocking phosphorylation at all four sites identified in this region of Elp1 leads to reduced Elongator function, with the S1209A mutant showing the greatest defect followed by S1198 S1202A and T1204A S1205A T1206A in decreasing order of severity, but with additivity between S1198 S1202A and T1204A S1205A T1206A leading to a defect as severe as that of S1209A. That acidic substitutions mimicking phosphorylation at each of the four sites conferred considerable Elongator function is consistent with the notion that phosphorylation at these sites functions positively for Elongator.

**Figure 3:**
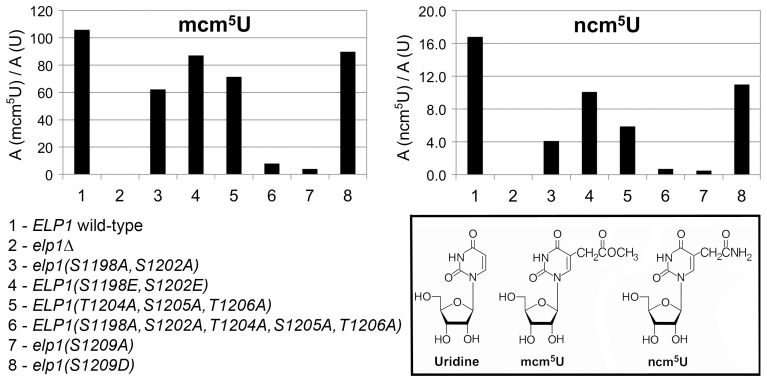
Quantitation of modified U34 nucleosides in tRNA from selected *elp1* mutants. Content of ncm^5^U and mcm^5^U in small RNA samples from the indicated yeast strains. In each case, A (modified U)/A (U) indicates that the modified nucleoside signal was normalized using the total uridine content to allow comparison of the different samples. Inset: chemical structures of uridine and its Elongator-dependent mcm^5^U and ncm^5^U derivatives.

To look directly at the tRNA wobble uridine modifications, we prepared tRNA from selected *elp1* mutant strains and used LC-MS analysis to quantitate the levels of modified U34 nucleosides. Fig. 3 shows that in the *elp1Δ* control strain, mcm^5^U and ncm^5^U were absent from tRNA as expected. The S1209A and quintuple S1198 S1202A T1204A S1205A T1206A strains showed almost no mcm^5^U or ncm^5^U, consistent with the major defect in Elongator-dependent wobble uridine modification indicated by the phenotypic assays (Fig. 2). The S1198A S1202A double mutant showed reduced levels of mcm^5^U and ncm^5^U consistent with a less severe Elongator defect, while strains with the S1198E S1202E double or S1209D single phosphomimic alleles, or with the triple T1204A S1205A T1206A allele, had levels of mcm^5^U and ncm^5^U intermediate between those of the S1198A S1202A mutant and *ELP1* wild-type. Taken together, the profiles for the ncm^5^ and mcm^5^ modification nucleosides are consistent with our phenotypic analyses (Fig. 2) and our data therefore support the notion that phosphorylation of Ser-1209 plays a major, positive role in Elongator-dependent tRNA modification and that phosphorylation at Ser-1198, Ser-1202 and Ser-1205/Thr-1206 makes a similar but partly redundant contribution to Elongator activity.

### Phosphorylation site mutations in Elp1 do not prevent Elongator assembly but lead to changes in association with Kti12 and with Hrr25 kinase

**Figure 4:**
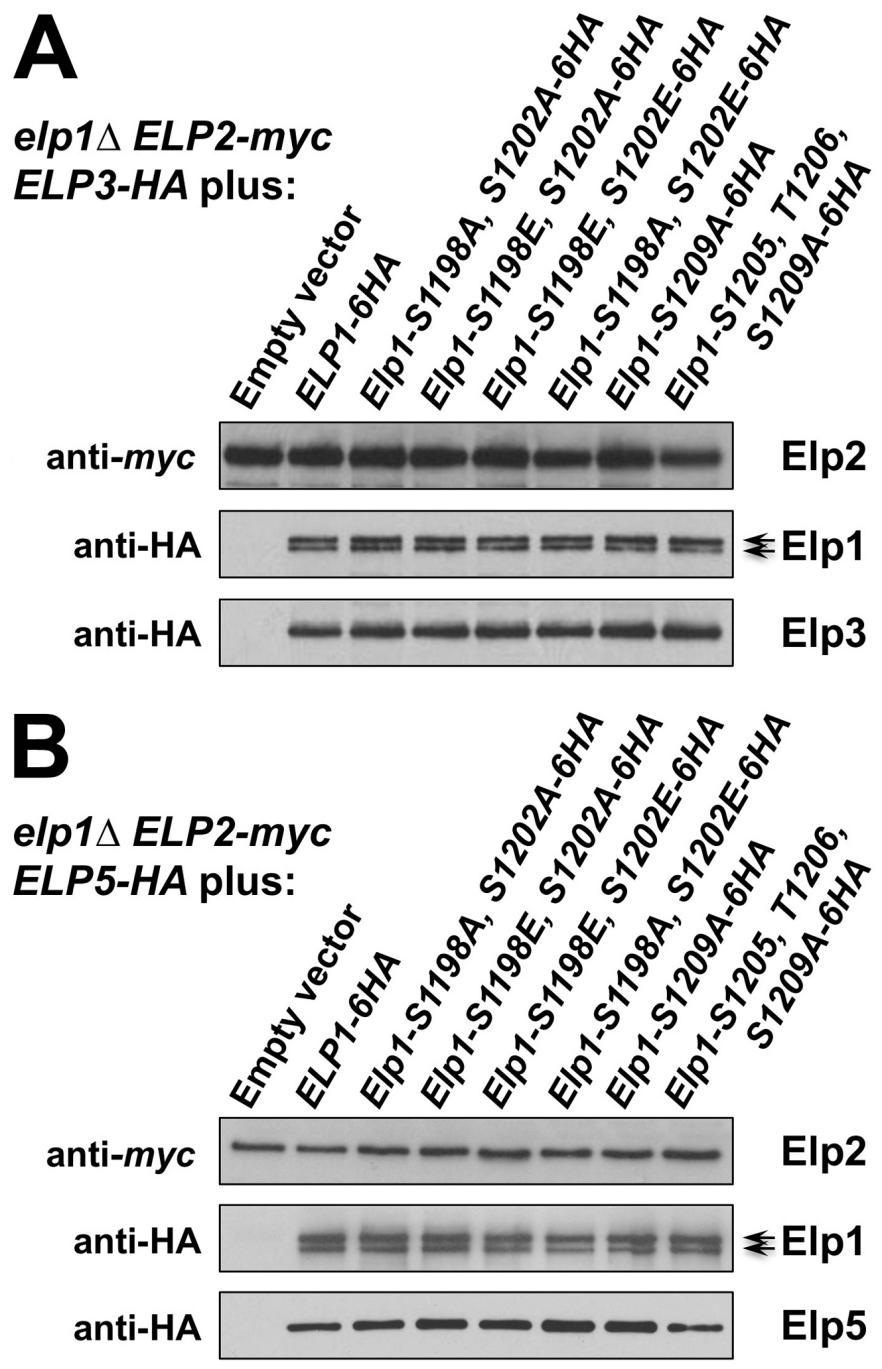
Elongator assembly is unaffected by a range of phosphorylation site mutations in Elp1. *elp1*Δ yeast strains expressing myc-tagged *ELP2* and either HA-tagged *ELP3* (A) or *ELP5* (B) were transformed with either empty vector or plasmids expressing the indicated *ELP1* alleles (Table S2). Elp2-myc was immunoprecipitated from extracts and immunoprecipitates were examined by Western blotting with anti-myc and anti-HA antibodies to detect co-immunoprecipitated Elp1, Elp3 and Elp5 as indicated. Note that Elp1 runs as a doublet as observed previously [84].

Phosphorylation site mutations that alter Elongator functionality could in principle do so by affecting assembly of the *holo*-Elongator complex, either because they interfere with assembly or because phosphorylation of these sites could regulate assembly of the Elongator complex. When myc-tagged Elp2 was used to immunoprecipitate Elongator from cell extracts, *elp1* phosphorylation site mutations at positions 1198, 1202, 1205, 1206 and 1209, alone or in combination, did not affect the recovery of Elp1, Elp3 and Elp5 in the Elp2 immunoprecipitates in comparison with strains where Elp1 was wild-type (Fig. 4). In particular, there was no evidence for any changes in the assembly of the complex when Elp1 carried the S1209A substitution, which is essentially null for Elongator function as discussed above. Thus similar co-immune precipitation of all four proteins was observed, regardless of whether these mutations affected Elongator functionality. Although we have not tested every mutant *elp1* allele constructed in this study, assembly of the Elp1-Elp2-Elp3 subcomplex and its association with the Elp4-Elp5-Elp6 subcomplex appear essentially normal, irrespective of the consequences of the mutations for Elongator functionality.

**Figure 5:**
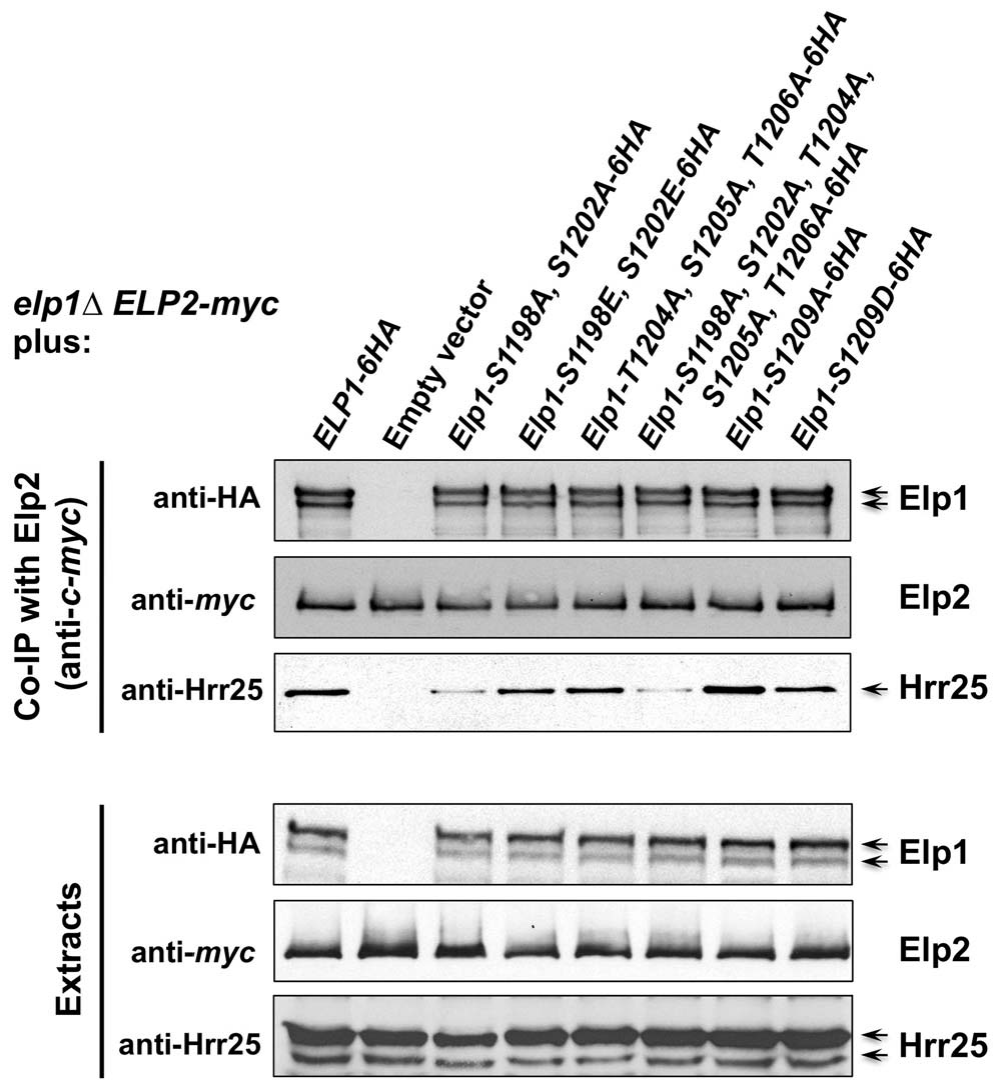
Phosphorylation site mutations in Elp1 lead to changes in Hrr25 association with Elongator. *elp1*Δ* ELP2-myc_3_ KTI12-HA_6_* yeast strains were transformed with either empty vector or plasmids expressing the indicated HA_6_-tagged *ELP1* alleles (Table S2). Elp2-myc was immunoprecipitated from extracts and immunoprecipitates were examined by Western blotting with anti-myc (to detect Elp2), anti-HA antibodies (to detect co-immunoprecipitated Elp1) and anti-Hrr25 (to detect co-immunoprecipitated Hrr25) as indicated. Western blots of the extracts before immunoprecipitation (lower panels) confirm similar levels of each protein in the different strains.

**Figure 6:**
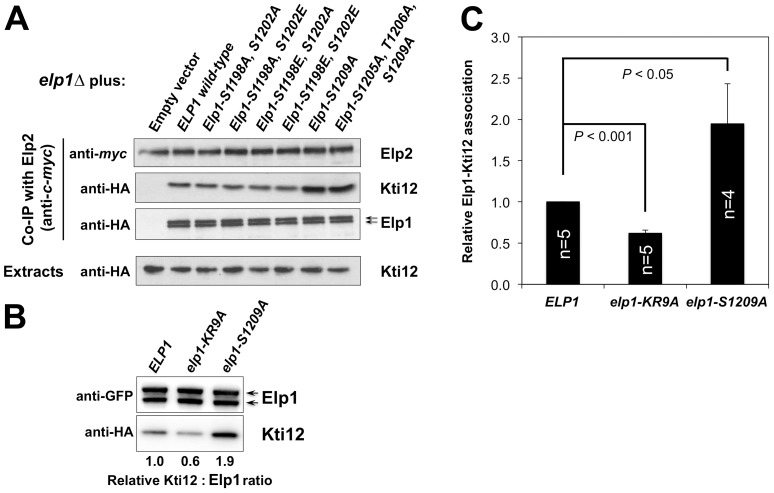
Phosphorylation site mutations in Elp1 lead to changes in Kti12 association with Elongator. (A) *elp1*Δ* ELP2-myc_3_ KTI12-HA_6_* yeast strains were transformed with either empty vector or plasmids expressing the indicated HA_6_-tagged *ELP1* alleles (Table S2). Elp2-myc was immunoprecipitated from extracts and immunoprecipitates were examined by Western blotting with anti-myc (to detect Elp2) and anti-HA antibodies (to detect co-immunoprecipitated Elp1 and Kti12) as indicated. Western blots of the extracts before immunoprecipitation (lower panels) confirm similar levels of each protein in the different strains. (B) Co-immune precipitation of HA-tagged Kti12 with GFP-tagged Elp1. Extracts from strains expressing GFP-tagged wild-type or mutant Elp1 from its genomic locus together with HA-tagged Kti12 were subjected to immune precipitation using GFP-trap and then the immune precipitates examined by Western blotting with anti-GFP (to detect Elp1) and anti-HA antibodies (to detect co-immunoprecipitated Kti12) as indicated. *elp1-KR9A*, a tRNA binding domain mutation that reduces Kti12 association [32], is shown as a control. (C) Quantification of co-immunoprecipitation efficiency shown in (B). Immunoprecipitation of HA-tagged Kti12 was quantified by densitometry of the HA tag signals and normalized using the Elp1–GFP signals across the indicated number of replicates (n), setting the value for the wild-type strain in each case to 1.0. Error bars represent the standard deviation of the mean and the significance of the differences was analyzed using a one-way ANOVA, showing a statistically significant, two-fold increase in the association of Kti12 with Elp1-S1209A.

Since Elongator interacts with both its accessory factor Kti12 and with Hrr25 kinase and because Elongator function requires both Kti12 and Hrr25 kinase activity [16], [37], [45], [46], we next used co-immunoprecipitation to examine the effect of selected phosphorylation site mutations on Elongator's association with Kti12 and Hrr25. Elongator complex in which Elp1 carries the double S1198A S1202A mutation showed reduced interaction with Hrr25 that was not seen with the corresponding phosphomimic allele (S1198E S1202E), but combining S1198A S1202A with T1204A S1205A T1206A did not further reduce interaction with Hrr25 (Fig. 5) despite the greater loss of Elongator function in the quintuple mutant. Conversely, mutation of Elp1 Ser-1209 to alanine led to enhanced interaction between Elongator and Hrr25 (Fig. 5). In all *ELP1* mutants tested, Elongator retained its ability to interact with Kti12, but the *elp1*-S1209A allele also led to enhanced Kti12 interaction (Fig. 6A). We examined the effect of the Elp1 S1209A substitution on Kti12 association in more detail by tagging the genomic copy of *ELP1* with GFP and carefully quantitating the recovery of HA-tagged Kti12 in strains expressing either the wild-type or mutant Elp1-GFP fusions. This confirmed that the S1209A mutation leads to enhanced Kti12 association: in comparison with wild-type Elp1, Elp1-S1209A was reproducibly associated with approximately twice as much Kti12 (Fig. 6B, C). Thus the S1198A S1202A and S1209A mutations have opposite effects on association with Hrr25 and the 1209A mutation enhances association with Kti12, suggesting that phosphorylation at these sites affects the interaction between Elongator and key proteins required for its functionality.

### Hrr25 directly phosphorylates Elp1 on functionally important sites

We next wished to identify the protein kinase(s) that mediate modification of the phosphorylation sites in the Elp1 carboxy-terminal domain that are important for Elongator function. To take an unbiased approach, we screened a library of 119 GST-protein kinase fusions [47] for their ability to phosphorylate Elp1 *in vitro* using TAP-purified Elongator as a substrate. Purified Elongator showed significant background phosphorylation specifically on Elp1 when incubated with [γ-^32^P]ATP in the absence of added kinase. However, five protein kinases were identified that could clearly phosphorylate Elp1 *in vitro* (Hrr25, Yck1, Yck2, Yck3 and Hal5: S2A Fig.). Given that Hrr25 interacts with Elongator [37], [48], [49] as seen in Fig. 5 and because Elp1 is already known to become hypophosphorylated in a *hrr25* mutant [37], it is an excellent candidate for an *in vivo* Elp1 kinase. Hrr25, Yck1, Yck2 and Yck3 all belong to the casein kinase I (CKI) family of protein kinases [50], but while *hrr25* mutants show clear defects in Elongator function [37], [38], Yck1, Yck2 and Yck3 are membrane associated via lipid modification [51], [52] and do not confer detectable zymocin resistance when the corresponding genes are deleted [38]. Thus we consider it unlikely that Yck1-3 mediate functionally important phosphorylation events on Elp1 *in vivo* and that they were identified in our *in vitro* screen due to shared substrate specificity with the related Hrr25 CKI isozyme. Preliminary analysis also failed to generate data supporting a role for Hal5 in Elongator function (S2B-S2C Fig.).

**Figure 7:**
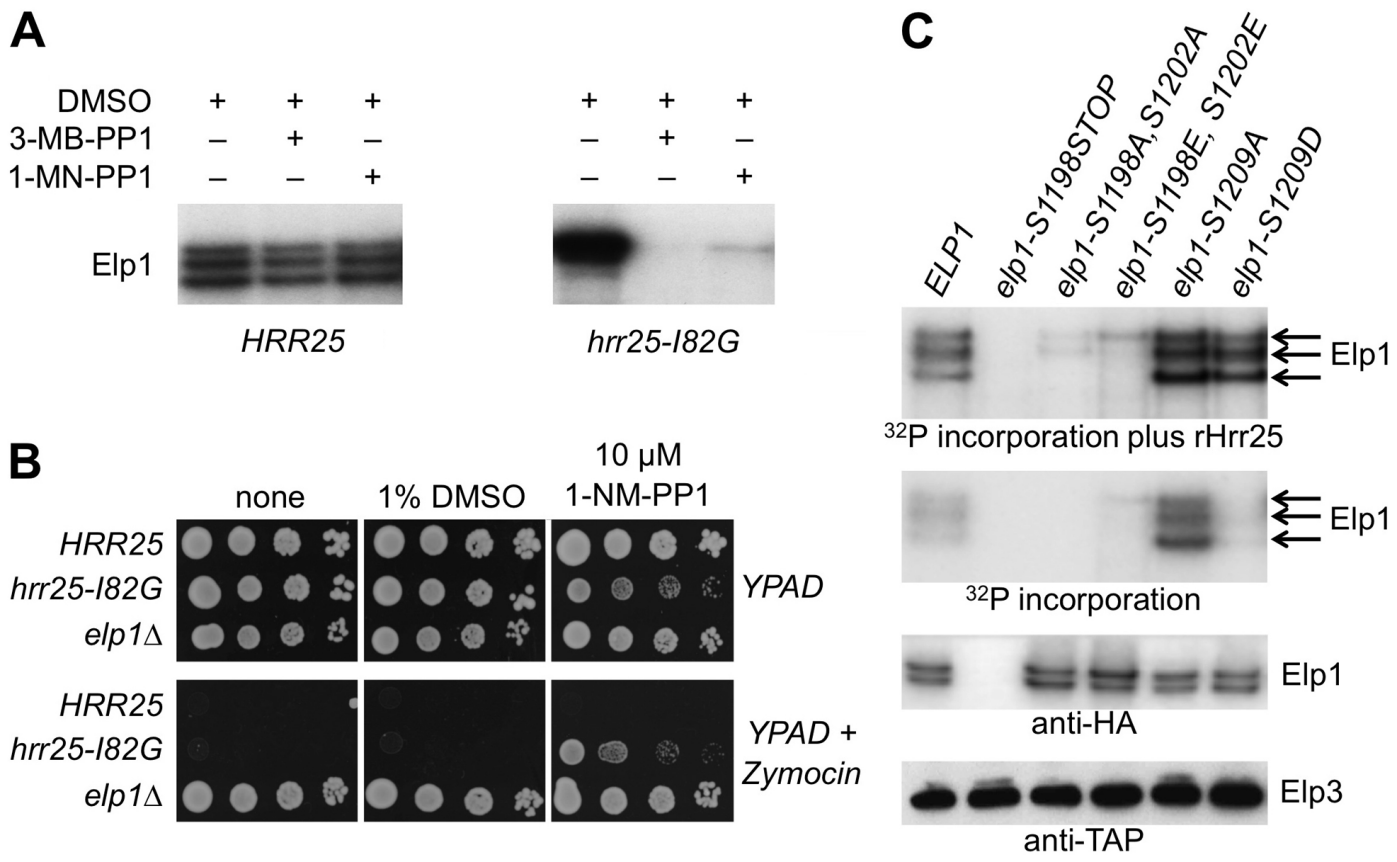
Purified Elongator shows Hrr25-dependendent phosphorylation that requires Ser-1198 and Ser-1202. (A) Elongator was purified using TAP-tagged Elp3 from either a *HRR25* wild-type or a *hrr25-I82G* analog-sensitive mutant strain and supplemented with [γ-^32^P]ATP to examine endogenous Elp1 phosphorylation in the presence of DMSO (drug vehicle), 1NM-PP1 or 3MB-PP1. Phosphorylated Elp1 was visualized by autoradiography following SDS-PAGE. (B) Equivalent 10-fold serial dilutions of *HRR25 ELP1*, *hrr25-I82G ELP1* and *HRR25 elp1Δ* yeast strains were plated out on YPAD agar with or without 1% (v/v) zymocin in the presence or absence of 10 µM 1NM-PP1 or an equivalent volume of DMSO (drug vehicle) as indicated and photographed after 2 days' growth at 30°C. (C) Elongator was prepared as in (A) from *elp1Δ ELP3*-TAP strains carrying plasmids encoding the indicated *ELP1* alleles and incubated with [γ-^32^P]ATP in the presence or absence of added recombinant Hrr25 (100 ng). Phosphorylated Elp1 was visualized by autoradiography following SDS-PAGE. Western blotting to detect Elp3-TAP and Elp1-6HA demonstrates equivalent recovery of Elongator from each strain.

In further support of a role for Hrr25 as a direct Elp1 kinase, we next showed that the Elp1 kinase activity present in affinity-isolated Elongator preparations was due to Hrr25. This made use of a yeast strain dependent on an ‘analog-sensitive’ *HRR25* allele (*hrr25*-I82G) in which the mutant Hrr25 kinase has acquired the capacity to be inhibited specifically by addition of the ATP analogs 1NM-PP1 or 3MB-PP1 [53]. When Elongator was isolated from the *hrr25*-I82G strain, the Elp1 phosphorylation observed upon incubation of the isolated complex with [γ-^32^P]ATP was blocked by addition of either 1NM-PP1 or 3MB-PP1 (Fig. 7A). This was in contrast to Elongator isolated from a control strain expressing wild-type *HRR25*, phosphorylation of which was refractory to these inhibitors. In fact the *hrr25*-I82G strain became zymocin resistant when grown in the presence of 1NM-PP1, emphasizing the positive role of Hrr25 kinase in Elongator's tRNA modification *in vivo* (Fig. 7B) and consistent with Elongator-minus phenotypes of *hrr25* mutants [37], [38].

To determine whether Hrr25 phosphorylation of Elp1 occurred at any of the functionally relevant sites that we had identified, Elongator containing wild-type or mutant forms of Elp1 was purified and tested for incorporation of phosphate from [γ-^32^P]ATP both in the absence and presence of recombinant Hrr25. Fig. 7C shows that even when reactions were supplemented with Hrr25, Elp1 in which the C-terminal region had been deleted was not phosphorylated. Elongator in which both Elp1 Ser-1198 and Ser-1202 had been replaced by either alanine or glutamate showed negligible incorporation of radiolabel in comparison with the Elp1 in wild-type Elongator. In contrast, Elongator in which Elp1 Ser-1209 was substituted with either alanine or aspartate showed high levels of ^32^P incorporation into Elp1 in the presence of recombinant Hrr25. Since *HRR25* is an essential gene we were unable to examine Ser-1209 phosphorylation with the anti-phophoserine-1209 antibody in the complete absence of Hrr25. However, in the kinase-dead *hrr25-3* mutant, which shows loss of Elongator function and absence of the phosphorylated isoform detected in wild-type cells by gel shift assay [37], a similar level of Ser-1209 phosphorylation to that seen in a wild-type strain was observed (S3 Fig.). Although more complex interpretations are possible, these observations are therefore most simply explained if Ser-1198 and Ser-1202 are major sites of Hrr25 phosphorylation and Ser-1209 is not a major site for modification by the kinase. In the absence of added Hrr25, Elongator complex containing Elp1-S1209A still showed high levels of Elp1 phosphorylation that were not seen with the corresponding aspartate substitution or when Elp1 was wild-type, although after addition of recombinant Hrr25 the level of Elp1 phosphorylation of the Elp1-S1209A and Elp1-S1209D complexes was similar as noted above (Fig. 7C). These data are consistent with the enhanced interaction of Elp1-S1209A-containing Elongator with Hrr25 that was seen by co-immune precipitation (Fig. 5).

To complement these experiments, the ability of recombinant Hrr25 to phosphorylate synthetic peptides corresponding to the C-terminal phosphorylated region of Elp1 was tested, using both mass spectrometry to identify phosphorylated residues in reactions with unlabeled ATP and by monitoring incorporation of ^32^P-phosphate in reactions containing [γ-^32^P]ATP. When a peptide containing Elp1 residues 1193-1213 was phosphorylated by recombinant Hrr25 *in vitro* and analyzed by mass spectrometry, three types of phosphopeptide were detected: monophosphorylated peptide modified on either Ser-1198 (S4A Fig.) or Ser-1202 (S4B Fig.), and diphosphorylated peptide modified on both these residues (S4C Fig.). These data therefore confirm the identity of Hrr25 as a protein kinase that can directly phosphorylate Elp1 on serine residues that are important for Elongator function, and are consistent with a model in which phosphorylation at one of these two sites may then favor phosphorylation of the second site. When all phosphorylatable residues apart from Ser-1198 and Ser-1202 were changed to alanines, dual phosphorylation on Ser-1198 and Ser-1202 was still seen, indicating that phosphorylation of these two positions was not dependent on phosphorylation of Ser-1205 or any other downstream residues.

**Figure 8:**
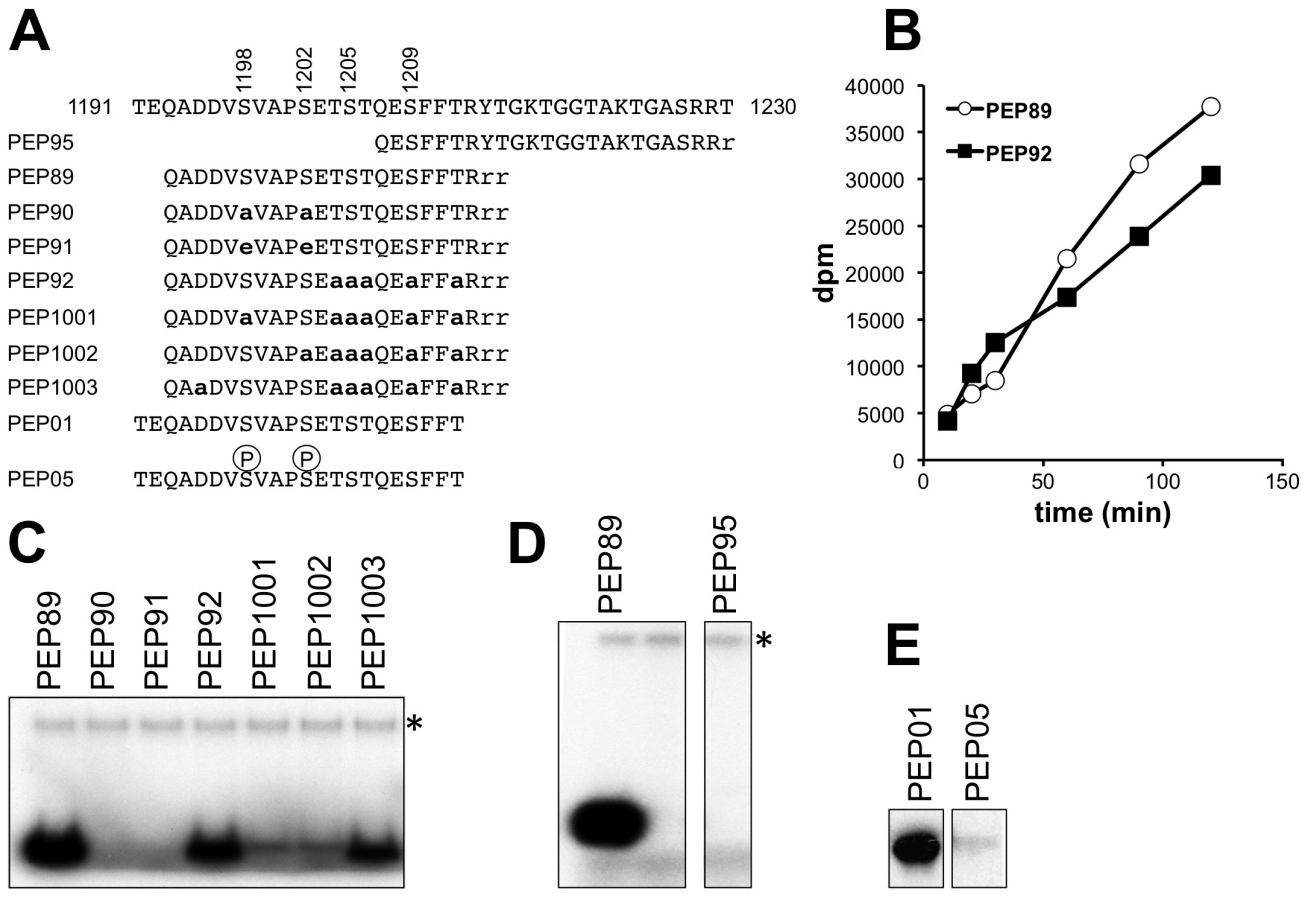
*In vitro* phosphorylation of peptides corresponding to the Elp1 C-terminal region by Hrr25. (A) Sequences of synthetic peptides used as substrates shown in alignment with Elp1 residues 1191-1230. Lower-case bold letters represent alanine and/or glutamate substitutions relative to the native Elp1 sequence. (B) Time course of Hrr25-dependent incorporation of ^32^P from [γ-^32^P]ATP into Peptides 89 and 92. (C-E) Incorporation of ^32^P from [γ-^32^P]ATP into peptides as assayed by SDS-PAGE and autoradiography. Note that in (D) and (E) two panels are shown in each case because some unnecessary intervening lanes were removed when preparing the figure. * indicates autophosphorylated recombinant Hrr25.

When the 1193-1213 peptide was incubated with Hrr25 together with [γ-^32^P]ATP, it readily incorporated radiolabelled phosphate (Fig. 8B and C; peptide 89). Although the related peptide in which all serine and threonine residues apart from Ser-1198 and Ser-1202 had been substituted by alanines was still an excellent Hrr25 substrate (Fig. 8C; peptide 92), incorporation of ^32^P at later times was reduced in comparison (Fig. 8B), consistent with the possibility of additional phosphorylation to the right of Ser-1202. Additional single alanine substitutions at either Ser-1198 or Ser-1202 in peptide 92 greatly reduced but did not completely prevent phosphorylation (Fig. 8C, peptides 1001, 1002). However, replacement of both Ser-1198 and Ser-1202 by either alanines or glutamates completely blocked phosphorylation of the peptide despite the presence of the five serine and threonine residues downstream (peptides 90 and 91). Another peptide encompassing Elp1 residues 1207-1229 was not phosphorylated at all following incubation with Hrr25 (Fig. 8D, peptide 95), supporting the notion that neither Ser-1209 nor any of the repeating threonine and serine shown in Fig. 1E can be directly phosphorylated by Hrr25 kinase. These data are therefore consistent with interdependence of Ser-1198 and Ser-1202 phosphorylation by Hrr25 and absence of Hrr25 phosphorylation on the downstream serines and threonines.

Since phosphorylation of specific Ser or Thr residues by CKIs such as Hrr25 is often primed by phosphorylation at another Ser or Thr residue 2-4 positions upstream [54], it was possible that Hrr25 might phosphorylate Ser-1205 once Ser-1202 had been modified, and that phosphorylation at Ser-1205 might then promote modification of Ser-1209. However, phosphomimic glutamate substitutions at 1198 and 1202, which supported essentially normal Elongator functionality *in vivo* (Fig. 2), also prevented phosphorylation of the 1193-1213 peptide on any other downstream site. Furthermore, phosphorylation of a related peptide (Elp1 1192-1212; Fig. 8E), which was also a good *in vitro* substrate for Hrr25, was also completely blocked for Hrr25 phosphorylation when synthesized with phosphoserine at the two positions corresponding to Elp1 Ser-1198 and Ser-1202. This again supports the model that Ser-1198 and Ser-1202 are the only major sites of Hrr25 phosphorylation and also indicates that it is unlikely that efficient priming of phosphorylation by Hrr25 on downstream residues, such as Ser-1205 and Ser-1209, occurs as a result of the upstream phosphorylation events at Ser-1198 and Ser-1202. Thus although these experiments do not as yet fully explain all the intricacies of Hrr25 phosphorylation in this region, taken together they nonetheless demonstrate that Hrr25 phosphorylates Elp1 directly on Ser-1198 and Ser-1202, two serine residues that we identified as functionally relevant *in vivo* phosphorylation sites.

## Discussion

### Identification of functionally relevant phosphorylation sites in Elp1

We have identified nine phosphorylation sites on the Elp1 subunit of yeast Elongator complex and based on the phenotypes of non-phosphorylatable and phosphomimic mutations, provide evidence that phosphorylation on four sites near the Elp1 C-terminus (Ser-1198, Ser-1202, Ser-1205/Thr-1206 and Ser-1209) plays a positive role in Elongator function. Previously, we showed that a *sit4* phosphatase mutant trapped Elp1 in a slower-migrating, hyperphosphorylated form whereas *hrr25* kinase mutations led to presence of just a faster-migrating, hypophosphorylated Elp1 isoform [37], [39]. Both types of mutation cause loss of Elongator function [37], [39], suggesting that dynamic phosphorylation and dephosphorylation of Elp1 is needed in functional Elongator and predicting that mimicking constitutive phosphorylation on at least some of the sites we have identified should be inhibitory. It is therefore surprising that all of the phosphomimic alleles we tested conferred significant Elongator function. Possibly the acidic substitutions do not fully mimic constitutive phosphorylation and thereby allow for substantial residual function, even though constitutive phosphorylation might inhibit Elongator. Thus while our alanine substitution mutants lend support to the idea that phosphorylation at the mapped sites functions positively for Elongator activity, we cannot rule out a requirement for dynamic phosphorylation/dephosphorylation at the sites we have identified. It is also possible that additional, inhibitory phosphorylation sites exist that were not found in our study. For example, although we could not detect phosphorylation of Thr-1212 *in vivo* or *in vitro*, both alanine and glutamate substitutions at this site caused severe loss of Elongator function (S5 Fig.), consistent with the notion that dynamic phosphorylation and dephosphorylation of this site could be important if it is phosphorylated *in vivo*.

### Hrr25 directly phosphorylates Elp1 on sites important for Elongator function

Despite conducting an unbiased, ‘kinome-wide’ screen to identify kinases responsible for Elp1 C-terminal domain phosphorylation, Hrr25, the yeast CKI that associates with Elongator and has already been implicated in Elongator function [37], [48], [49] was the sole convincing candidate to be identified. Through several lines of evidence, we have now established that Hrr25 directly phosphorylates Elp1 on two *in vivo* phosphorylation sites that we have mapped and shown to be relevant for Elongator function: Ser-1198 and Ser-1202. Thus purified Elongator shows Hrr25-dependent phosphorylation that requires the presence of these two residues, while peptides derived from the Elp1 C-terminal region show direct, interdependent phosphorylation by recombinant Hrr25 on Ser-1198 and Ser-1202. Conversely, we found no evidence for direct phosphorylation of Ser-1209 by Hrr25 using three different synthetic peptides containing Ser-1209, or using purified Elongator complex in which Ser-1198 and Ser-1202 were mutated but Ser-1209 was intact.

Two types of consensus phosphorylation site have been proposed for CKIs: pS [X]_1-3_ [**ST**] and [DE]_2-4_ [X]_0-2_ [**ST**], where pS indicates an upstream phosphoserine residue that is needed to prime phosphorylation at the downstream Ser or Thr (shown in bold and underlined) but which can be substituted by an acidic patch in the second class of motif [54]. Ser-1198 conforms to the latter type of motif and is closely related to other mapped Hrr25 phosphorylation sites [55]-[57] in yeast proteins (S6 Fig.). In contrast, Ser-1202 matches the former motif, suggesting that priming-independent phosphorylation at Ser-1198 by Hrr25 might then prime phosphorylation by Hrr25 at Ser-1202 and predicting that Ser-1198 should still be efficiently phosphorylated when Ser-1202 is replaced by alanine. However, the latter mutation largely blocked phosphorylation at Ser-1198 in our *in vitro* peptide kinase assays and instead we observed interdependent phosphorylation at these two residues. Furthermore, replacement of one of the two upstream aspartate residues with alanine did not obviously interfere with phosphorylation of Ser-1198 and Ser-1202. Thus although the sites are direct targets of Hrr25 and match the accepted consensus for CKI phosphorylation, dependency of Ser-1202 phosphorylation on prior Ser-1198 modification remains to be demonstrated. Similarly, Ser-1205 and Ser-1209 both fit the pS [X]_1-3_ [**ST**] consensus and might be modified once Ser-1202 phosphorylation has occurred, and yet we could not demonstrate Hrr25-dependent incorporation of phosphate at these sites even using a peptide where the upstream sites were already phosphorylated. Given the known substrate specificity of CKIs, it is therefore surprising that we can find no evidence for such priming of phosphorylation at Ser-1205 and Ser-1209 following modification of Ser1202. Furthermore, since the repeating pattern of phosphorylatable residues shown in Fig. 1E also fits the pS [X]_1-3_ [**ST**] motif we are even more surprised that we failed to detect phosphorylation of these sites either *in vivo* or *in vitro*.

In spite of the strong evidence we have provided that Hrr25 is a direct Elp1 kinase, inability to detect direct phosphorylation of either the key residue Ser-1209 or of Ser-1205/Thr-1206 by Hrr25 implies that at least one additional Elp1 kinase is involved in Elp1 phosphorylation. While these sites might become better substrates for Hrr25 in the context of fully assembled Elongator complex rather than within synthetic peptides, the fact that we detect normal levels of Ser-1209 phosphorylation in a *hrr25* mutant that is defective for Elongator function strongly supports the involvement of a different kinase at Ser-1209.

### Elp1 phosphorylation may regulate Elongator's interaction with Kti12 and Hrr25

Although all phosphorylation site mutants examined showed essentially normal assembly of the Elongator complex and retained the ability to interact with the accessory protein Kti12, the S1209A mutation enhanced association of Kti12 with Elongator in comparison with cells expressing either the wild-type protein or the S1198A S1202A mutant. Kti12 stoichiometry is important – either too much or too little interferes with Elongator function [39], [43], [45] – and thus Ser-1209 phosphorylation may regulate Elongator's interaction with its accessory protein. Furthermore, the S1209A mutation caused increased Hrr25 association with Elongator whereas the double S1198A 1202A mutation led to reduced Hrr25 association. Thus Hrr25 not only directly phosphorylates Elp1 but may also regulate its own interaction with Elongator through doing so. Although the S1209A single and S1198A S1202A T1204A S1205S T1206A quintuple mutants both conferred strong loss of Elongator function, their differing effects on Hrr25 association implies that they are defective for different reasons – phosphorylation at S1198A S1202A may stabilize Hrr25 binding whereas phosphorylation at Ser-1209 may be required to displace the kinase. Since the interaction between Hrr25 and Elongator is dependent on Kti12 [37] it is also possible that the enhanced interaction of both proteins with Elongator seen when Elp1 cannot be phosphorylated on Ser-1209 are directly related.

Intriguingly, enhanced interaction of both Kti12 and Hrr25 with the Elongator complex is also seen in *hrr25* mutants that cause Elp1 phosphorylation defects and zymocin resistance [37 and S7 Fig.]. However, these properties of *hrr25* mutants are more similar to those of the *elp1*-S1209A mutant, which is mutated at a site apparently not directly phosphorylated by Hrr25, rather than mirroring the properties of the *elp1*-S1198A, S1202A mutant that removes the only sites in Elp1 that we have shown to be direct Hrr25 targets. This suggests an as yet undiscovered connection between phosphorylation at Ser-1209 and Hrr25 kinase. Regardless of this, our data nonetheless indicate that Elp1 phosphorylation by Hrr25 and other kinases could modulate the interaction between Elongator and Kti12.

### What type of role does Elp1 phosphorylation play in Elongator function?

Although there is clearly more to learn about the role of phosphorylation in Elongator function, two types of model can be proposed. On the one hand, phosphorylation might regulate Elongator, turning its wobble uridine modification activity up or down in response to growth conditions and the demand for protein synthesis, or perhaps in response to cellular stresses. Given that the translation of some mRNAs is particularly dependent on wobble base modification and Elongator function [58]-[60] and that tRNA modifications (including Elongator-dependent ones) oscillate during the cell cycle and in response to stress signals [61], [62], this raises the interesting possibility that Elongator may be part of a translational control mechanism functioning through tRNA modification. Our proposal that Elp1 phosphorylation operates in a largely positive sense for Elongator, based on the properties of the phosphorylation site mutants that we have examined, is consistent with such a regulatory role. The Elp1 kinase Hrr25 is needed for a wide range of cellular functions [49], [56], [57], [63]-[65] that do not appear to provide a clear insight into the signals that might regulate Elp1 phosphorylation. However, the Hrr25 requirement for full functionality of two different components of the translation machinery - wobble uridine-containing tRNAs [37, this work] and ribosomes [49], [57] - might reflect a role in regulation of the cell's capacity for protein synthesis. In addition, both *hrr25* mutants [66] and Elongator-deficient yeast [17] are sensitive to hydroxyurea and methyl methanesulfonate. Since efficient translation of the ribonucleotide reductase gene *RNR1* requires mcm^5^-modified tRNAs [62], [67], Elp1 phosphorylation may also be linked to the known role of Hrr25 in expression of genes such as *RNR2* and *RNR3* in response to DNA damage [66].

Alternatively, Elp1 phosphorylation might be dynamic, with sequential phosphorylation and dephosphorylation of specific residues driving the biochemical mechanism through which Elongator carries out the tRNA modification reactions. Such a dynamic role might operate through modulation of Elongator's interaction with factors such as Kti12 or with tRNA. It is intriguing in this context that the C-terminal phosphorylated region in Elp1 is immediately adjacent to a basic region that binds specifically to tRNA and that mutation of the tRNA binding domain leads to reduced interaction with Kti12 [Fig. 6C and ref. 32]. Thus phosphorylation of Elp1 could potentially influence how Elp1 interacts with tRNA, perhaps through interaction between the acidic phosphate groups and the basic residues present in the tRNA binding domain.

In conclusion, while there is still much to learn about Elp1 phosphorylation and its involvement in Elongator function, our work clearly demonstrates the importance of at least four in vivo phosphorylation sites in the C-terminal domain of Elp1 for Elongator-dependent tRNA wobble uridine modification, shows that Hrr25 kinase directly modifies two of these sites and provides evidence that phosphorylation regulates the association between Elongator and both its accessory protein Kti12 and its kinase Hrr25.

## Materials and Methods

### Yeast strains and plasmids

Basic yeast methods, growth media, and routine recombinant DNA methodology were performed as previously described [68], [69]. All plasmids used in this study are listed in Table S2 and yeast strains are listed in Table S3. To generate yeast strains dependent on wild-type or mutant forms of *ELP1*, *ELP1* was first deleted from BY4741 and WAY008 (*ELP3-TAP*) using pFA6a-*KanMX6* and pCORE-UH deletion cassettes to obtain the *elp1Δ* knockout mutant strains WAP034 and WAY037, respectively. Wild-type or mutant *ELP1* variants carried on the low-copy plasmid YCplac111-*ELP1*-6HA (Table S2) were then introduced into these *elp1Δ* strains as the sole source of Elp1. The mutant versions were made by either site directed mutagenesis (Qiagen QuikChange) using YCplac111-*ELP1*-6HA as template, or by replacing the relevant region using standard cloning procedures and synthetic DNA carrying the desired mutations. The majority of plasmids made using the latter approach were produced by DNA2.0, Inc (Menlo Park, CA, USA). All *elp1* mutants made by site-directed mutagenesis were verified by DNA sequencing of the entire *ELP1* ORF plus approximately 200 base pairs upstream and downstream. When mutations were introduced by cloning, the region that had been replaced was sequenced to exclude the possibility of gene synthesis errors. The wild-type and mutant versions of YCplac111-*ELP1*-6HA were transformed into WAY034, WAY037, or *elp1Δ::KanMX6 ELP2-myc_3_ strains* carrying additional epitope-tagged components (Table S3) for phenotypic screening, Elongator-complex purification or Western blot analysis, respectively. pFA6a-CTAP4-*HIS3MX6* was made by ligating the 1322 bp *Bgl*II-*Pme*I fragment from pFA6a-3HA-*HIS3MX6* to the 3603 bp *Bgl*II-*Pme*I fragment from pFA6a-CTAP4-NatMX6 [70] such that the *NatMX* marker was replaced by *HIS3MX*. Using this template, a C-terminal TAP tag was added to *ELP1* in RL-343-F0 and RL-343-F1 (Table S3) using standard one-step tagging methodology [71]. *sit4Δ::LEU2* and *kti12Δ::LEU2* knockouts were made as previously described [39], [43], as was introduction of *elp1*Δ*::KanMX6* in strains used for co-immune precipitation with *myc*-tagged Elp2 [16]. Construction of *ELP1-HA6::KlTRP1* utilized the pYM3 tagging plasmid and S2/S3 primer set described by Knop et al. [72].

### Purification of TAP-tagged Elongator

For phosphorylation site mapping, Elongator complexes were purified from WAY009, WAY010, WAY011, WAY-H-P1T and WAY-Has-P1T (Table S3) by two-step tandem affinity purification of Elp1-TAP from 2-12 liter cultures in YPAD medium grown to OD_600_ 1.0-1.5, as described previously [73]. All purifications were done in the presence of PhosSTOP and Complete protease inhibitor cocktail (Roche). Elongator complexes in which Elp1 contained phosphorylation site mutations were similarly purified from WAY037 (*elp1Δ::pCORE-UH ELP3-TAP::HIS3MX*: Table S3) harboring YCplac111-ELP1-6HA or its mutant derivatives (Table S2), growing cells as above but in SCD-Leu to select for retention of the plasmid.

### Phosphorylation site mapping

Elongator protein preparations were digested in solution with Trypsin (Trypsin Gold, mass spectrometry grade; Promega V5280). The resulting peptides were cleaned over Hypersep C18 columns (Thermo Scientific) to remove buffer contaminants, eluting the peptides in 70% acetonitrile, 0.1% trifluoroacetic acid (TFA). Phosphopeptide enrichment was done in a two-step procedure using a Hypersep SCX column (Thermo Scientific) followed by TiO_2_ enrichment of mono-phosphorylated peptides from the SCX flow-through. SCX binding and washing buffer contained 10 mM KH_2_PO_4_, 25% acetonitrile (pH 3.0) and the elution buffer contained 10 mM KH_2_PO_4_, 25% acetonitrile, 350 mM KCl (pH 3.0). The flow-through was reduced in volume to ∼100 µl, supplemented with 100 µl 80% acetonitrile/2% TFA containing 200 mg/ml 2,5-dihydroxybenzoic acid (DHB), pH 2.0, then the sample applied to 5 micron Titansphere TiO_2_ beads (GL Sciences) and rotated gently at room temperature for 1 h. The beads were washed twice with 80% acetonitrile/2% TFA, 200 mg/ml DHB (pH 2.0), then 3 times with 80% acetonitrile/2%TFA (pH 2.0), before eluting with 60 µl 400 mM NH_4_OH (pH 11.0) and then supplementing with 2 µl of 100% formic acid. All phosphopeptide fractions were cleaned over C18 and submitted for mass spectrometry in 0.1% TFA.

Phosphopeptide fractions were analyzed by LC-MS/MS using a Dionex U300 system (Dionex California) with a PepMap C18 column coupled to either an Orbitrap XL or Orbitrap Velos (Thermo Fisher Scientific). Peptides were eluted using a 45 min 5%-90% acetonitrile gradient, sequencing the top 5 most intense ions with the following settings: CID, FTMS 335-1800 Da, 60,000 resolution, MS/MS charge state 1+ rejected,>2+ accepted. Peak picking, recalibration and peptide mass fingerprinting was done using MaxQuant software [74], [75], searching masses against the *Saccharomyces* Genome Database orf_trans_all database (January 5^th^ 2010 release) with 10 ppm MS error, ≤ 2 missed cleavages, Trypsin/P enzyme, variable modifications set to Acetyl (protein N-term), Oxidation (M), Phospho (ST) and Phospho (Y) and a fixed modification of Carbomidomethyl (C). The MS/MS tolerance was set to 0.5 Da and the false discovery rate for site, protein and peptide identification was set to 0.01. All phosphorylation sites identified in this way were verified by hand annotating the spectra. The analysis was done on four biological replicates of the finally optimized SCX-TiO_2_ protocol.

### Anti-Elp1 PhosphoSer-1209 antibody

An anti-Elp1 phosphoSer-1209 antibody was raised in a rabbit by BioGenes GmbH, using a peptide antigen (H_2_N-CTSTQE-pS-FFTRY-CO.NH_2_, where pS indicates phosphoserine) and their standard procedures (see http://www.biogenes-antibodies.com). The phosphospecific fraction of the final bleed was purified by serum depletion using immobilized non-phosphorylated peptide (H_2_N-CTSTQESFFTRY-CO.NH_2_), then phosphospecific antibodies were isolated from the depleted serum using immobilized phosphopeptide. The purified phosphospecific antibody fraction was tested for phosphospecificity by Western blotting, showing loss of signal when protein extracts were prepared from an *elp1-1209A* mutant and following competition with the phosphopeptide. The antibody was stored at -20°C in 50% (v/v) glycerol and used at a 1 in 3000 dilution. A small excess of the non-phosphorylated peptide was added to the primary dilution before use to prevent any cross-reaction with the non-phosphorylated epitope.

### Phenotypic assays

To test the effect of extracellular zymocin on wild-type and mutant strains, killer-eclipse assays were performed as described previously [76] using the *K. lactis* zymocin producer strain NCYC1368 and YPD plates prepared using Kobe I agar (Roth 5210). To test the effect of intracellular expression of zymocin's γ subunit on growth, wild-type and mutant *elp1* strains were transformed with pAE1, which expresses the γ subunit from the galactose-inducible *GAL1* promoter [43]. The response to zymocin γ-toxin induction was monitored on galactose plates after 3-4 days at 30°C. *SUP4* suppression efficiency was measured following integration of pSB3 in single copy at the *his3Δ0* locus and then monitoring growth on SCD-Leu-Ura after 3 days at 30°C as described previously [32]. To test the effect of Hrr25 inhibition on Elongator function, strains dependent on w.t. or analog-sensitive Hrr25 were grown overnight in YPAD medium, diluted to 1.0 A_600_/ml and then 10-fold dilutions were spotted onto YPAD agar with or without 1%(v/v) zymocin and containing 10 µM 1NM-PP1 or an equivalent volume of DMSO as drug vehicle control. Growth was documented after 2 days growth at 30°C. Zymocin was prepared using cell-free culture medium from *K. lactis* NCYC1368 which had been grown at 30°C for 2 days in YPAD medium, by 50-fold concentration using Amicon Ultra-15 Centrifugal Filter Units (Millipore) followed by sterilization by filtration.

### Relative quantitation of tRNA wobble uridine modifications by HPLC-DAD-MS/MS analysis

*elp1Δ* yeast strains containing YCplac111-*ELP1*-HA6 or its mutant derivatives were grown in SCD-Leu to select for the plasmid and then total tRNA was prepared by RNA extraction and LiCl precipitation as described previously [32]. Prior to LC-MS/MS analysis, 5 µg of each tRNA sample were digested into nucleosides by incubation at 37°C for 2 h in the presence of 1/10 volume of 10× nuclease P1 buffer (0.2 M NH_4_OAc pH 5.0, ZnCl_2_ 0.2 mM), 0.3 U nuclease P1 (Sigma Aldrich, Munich, Germany) and 0.1 U snake venom phosphodiesterase (Worthington, Lakewood, USA). Next, 1/10 volume of 10× fast alkaline phosphatase buffer and 1 U fast alkaline phosphatase (Fermentas, St. Leon-Roth, Germany) were added, and samples were incubated for additional 60 min at 37°C. The digested tRNA samples were analyzed on an Agilent 1260 HPLC series equipped with a diode array detector (DAD) and a triple quadrupol mass spectrometer (Agilent 6460). A Synergy Fusion RP column (4 µm particle size, 80 Å pore size, 250 mm length, 2 mm inner diameter) from Phenomenex (Aschaffenburg, Germany) was used at 35°C column temperature. The solvents consisted of 5 mM ammonium acetate buffer adjusted to pH 5.3 using acetic acid (solvent A) and pure acetonitrile (solvent B). The elution was performed at a flow rate of 0.35 ml/min using a linear gradient from 0% to 8% solvent B at 10 min, 40% solvent B at 20 min and 0% solvent B at 23 min. For additional 7 min, the column was rinsed with 100% solvent A to restore the initial conditions. Prior to entering the mass spectrometer, the effluent from the column was measured photometrically at 254 nm by the DAD for detection of the 4 canonical nucleosides. The triple quadruple mass spectrometer, equipped with an electrospray ion source (Agilent Jet Stream), was run at the following ESI parameters: gas (N_2_) temperature 350°C, gas (N_2_) flow 8 L/min, nebulizer pressure 50 psi, sheath gas (N_2_) temperature 350°C, sheath gas (N_2_) flow 12 L/min and capillary voltage 3000 V. The MS was operated in positive ion mode using Agilent MassHunter software and modified nucleosides were monitored by multiple reaction monitoring (dynamic MRM mode). Identification of ncm^5^U and mcm^5^U peaks was performed as described previously [77]. Peak areas were determined employing Agilent MassHunter Qualitative Analysis Software. In the case of the major nucleosides, peak areas were extracted from the recorded UV chromatograms in order to avoid saturation of the mass signals. For inter-sample comparability of the detected modifications, the peak areas of the modified nucleosides were normalized to the UV peak area of uridine.

### Western blotting and co-immune precipitation

Detection of tagged proteins used anti-TAP (Thermo Scientific, CAB1001), anti-myc and anti-HA antibodies (Roche) and was performed as previously described [16], [45]. Protein concentrations were determined using Quick StartTM Bradford Protein Assay (BioRAD) [78] and checked with anti-Pfk1 antibodies recognizing yeast Pfk1 (1:50,000, kindly provided by Dr. J. Heinisch) or anti-Cdc19 serum (1:10,000, kindly provided by Dr. J. Thorner) so as to ensure equivalent loadings. For detection of the Hrr25 kinase in total yeast extracts and in immune precipitates, a generic anti-Hrr25 antibody [64] was used (1:10,000 dilution). Antibody cross-linking to Dynabeads M-270 Epoxy (Invitogen), preparation of protein extracts and immune precipitation were performed according to the manufacturer's instruction and as described previously [16], [79]. In general, 1 µg of antibody coupled to Dynabeads was used per 1 mg total cell extract in B60 buffer.

### Identification of protein kinases that phosphorylate Elp1

All 119 GST-protein kinases constructs in the library described by Zhu et al. [47] were expressed in yeast and purified as described previously [80]. Yeast Elongator complex was isolated as described in the main paper and used as a substrate for the GST-kinases, which were initially assayed in 23 pools of 5 kinases under similar conditions to those described below. Reactions were separated by SDS-PAGE and radiolabelled Elp1 detected by autoradiography. Eight out of the 23 pools showed phosphorylation occurred above the background level observed due to co-purification of Hrr25 with Elongator (see Results). From these eight pools, 40 individual kinases were individually screened for their ability to phosphorylate Elp1. Reactions in which Elongator was omitted controlled for any radiolabeled bands that co-migrated with Elp1 and were therefore due to kinase autophosphorylation or co-purified kinase substrates.

### *In vitro* protein kinase assays

*E. coli* BL21 (DE3) pLysS (Novagen) was transformed with pTrcHis-*HRR25* for overexpression and purification of His_6_-Hrr25 using HisPur cobalt resin (Thermo Scientific) according to the manufacturer's instructions. To analyze Elp1 phosphorylation *in vitro*, purified wild-type or mutant Elongator complex (1 µg) was incubated for 30 min at 30°C in 20 µl P-buffer (50 mM HEPES-KOH (pH 8.0), 5 mM MgCl_2_, 50 mM KCl) containing 100 µM ATP and 0.5 µCi of radiolabelled [γ-^32^P]ATP (3000 Ci/mmol), in the absence or presence of purified recombinant His_6_-Hrr25 (1 µg). Phosphorylation reactions were stopped by the addition of NuPAGE 4× LDS Sample Buffer (Life Technologies) and heated for 5 min at 95°C. Samples were run on a NuPAGE (4-12%) polyacrylamide/Bis-Tris SDS gel (Life Technologies) at 200 V for 1 h. Gels were stained with Bio-Safe Coomassie (BioRad), dried and subjected to autoradiography. To examine the effect of chemical inactivation of yeast Hrr25 on Elp1 phosphorylation in purified Elongator complex, Elongator was purified as above from strains WAY-H-P1T (wild-type *HRR25*) and WAY-Has-P1T (allele-sensitive *hrr25* I82G) and assayed as described above in the presence or absence of 10 µM 3-MB-PP1 or 1-NM-PP1.

Time course phosphorylation reactions of synthetic peptides were carried out in 150 µl P-buffer containing 500 pmol synthetic peptide (Biomatik), 400 pmol recombinant His_6_-Hrr25, 1 mM ATP and 10 µCi [γ-^32^P]ATP (6000 Ci/mmol) and incubated at 30°C. Samples (5 µl) were collected at time intervals, spotted on Whatman Grade P81 Ion Exchange Cellulose Chromatography Paper, washed 3 times in 1% phosphoric acid, dried and quantitated by liquid scintillation counting [81]. To visualize phosphorylated peptides, 10 µl reactions were assembled at the above described stoichiometry, incubated for 30 min at 30°C and then terminated by addition of 4× SDS loading dye and heating at 95°C for 5 min. After separation by SDS-PAGE at 200 V using a NuPAGE 12% polyacrylamide/Bis-Tris gel (Life Technologies) with MES running buffer (50 mM MES, 50 mM Tris-base, 0.1% SDS, 1 mM EDTA, pH 7.3), phosphorylated peptides were visualized by autoradiography. For mass spectrometric analysis of phosphorylated peptides, 150 µl reactions were conducted as above but omitting the radiolabelled ATP.

## Supporting Information

S1 Fig Mapping *in vivo* phosphorylation sites in yeast Elp1. Representative MS/MS spectra are shown in which diagnostic B (green) and Y (blue) ions are mapped onto the corresponding peptide sequence. S(Ph), phosphoserine; M(Ox), oxidized methionine. (A) Phosphorylation on Ser-529 within the Elp1 peptide 521-531, parent MH^2+^ ion m/z 675.29. (B) Dual phosphorylation on Ser-539 and Ser-551 within the Elp1 peptide 532-552, parent MH^3+^ ion m/z 817.41. (C) Phosphorylation on Ser-636 within the Elp1 peptide 624-652, parent MH^3+^ ion m/z 1063.46. (D) Phosphorylation on Ser-828 within the Elp1 peptide 828-844, parent MH^2+^ ion m/z 1051.44. (E) Phosphorylation on Ser-1198 within the Elp1 peptide 1180-1213, parent MH^3+^ ion m/z 1314.56. (F) Phosphorylation on Ser-1202 within the Elp1 peptide 1180-1213, parent MH^2+^ ion m/z 1314.56. This spectrum represents a mixture of monophosphorylated isoforms of the peptide: * denotes peaks consistent with Ser-1198 phosphorylation, # indicates peaks consistent with Ser-1205 phosphorylation and ▪ indicates an ion consistent with Thr-1206 phosphorylation. (G) Phosphorylation on Ser-1205 or Thr-1206 within the Elp1 peptide 1180-1213, parent MH^2+^ ion m/z 1314.56.
(TIF)

S2 Fig Identification of Elp1 kinases. (A) Phosphorylation of yeast Elongator by yeast protein kinases. Elongator was purified from yeast using TAP-tagged Elp3 and incorporation of ^32^P from [γ-^32^P]ATP in response to GST-kinase fusions identified from the GST-kinase library of Zhu *et al*. [47]. Background incorporation of ^32^P in the absence of added kinase is due to co-purification of Hrr25 with affinity-purified Elongator complex as shown in the main paper. Elp1 runs as a doublet, the faster-migrating component of which is truncated at the amino-terminus [84]. (B) Relatedness of Hal5 to Kkq8 and Sat4. A standard BLAST search (http://blast.ncbi.nlm.nih.gov/Blast.cgi) of the yeast proteome with Hal5 using the default parameters indicates that Kkq8 and Sat4 are its closest relatives within the yeast kinome. (C) Deletion of *HAL5*, either alone or in combination with *KKQ8* and/or *SAT4*, fails to confer zymocin sensitivity by eclipse assay.
(TIF)

S3 Fig Elp1 shows similar levels of Ser-1209 phosphorylation in wild-type and *hrr25-3* mutant strains. Elp1 phosphorylation in samples of extract containing equivalent amounts of protein was monitored by Western blot analysis using the phosphoSer-1209 phosphospecific antibody.
(TIF)

S4 Fig Mapping of phosphorylation sites following *in vitro* phosphorylation of a peptide corresponding to Elp1 residues 1193–1213. MS/MS spectra are shown in which diagnostic B (green) and Y (blue) ions are mapped onto the corresponding peptide sequence. S(Ph), phosphoserine. (A) Phosphorylation on Ser-1198, parent MH^3+^ ion m/z 889.08 corresponding to monophosphorylated peptide. * denotes peaks consistent with phosphorylation on Ser-1202, suggesting that this is a mixed spectrum. (B) Phosphorylation on Ser-1202, parent MH^3+^ ion m/z 889.08 corresponding to monophosphorylated peptide. * indicates peaks consistent with phosphorylation of Ser-1198, suggesting that this is a mixed spectrum. (C) Phosphorylation on Ser-1198 and Ser-1202, parent MH^3+^ ion m/z 925.73 corresponding to diphosphorylated peptide. * denotes peaks consistent with one phosphate on Ser-1202 and a second phosphate to the right, most likely on Ser-1205.
(TIF)

S5 Fig Neither alanine nor glutamate substitutions at Elp1 Thr-1212 support Elongator function. All strains were based on WAY034 transformed with YCplac111 (empty vector; *elp1*Δ), YCplac111-*ELP1*-6HA (*ELP1* wild-type) or its Thr-1212 mutant derivatives as indicated. Zymocin sensitivity was measured by eclipse assay (see Fig. 1), testing samples of cells at either 1.0 or 0.1 OD_600_/ml following growth in SCD-Leu medium to select for the plasmids.
(TIF)

S6 Fig Alignment of Hrr25 phosphorylation sites. Alignment of the Elp1 Ser-1198 phosphorylation site with mapped Hrr25 phosphorylation sites in Tif6 [57], Atg19 [56] and Mam1 [55]. Sites known to be phosphorylated directly by Hrr25 are shown in red. Residues surrounding the mapped phosphorylation sites are highlighted in yellow (acidic), grey (small hydrophobic) or green (polar). In each case the phosphorylated residue is preceded by two acidic and one small hydrophobic residue and followed by two small hydrophobic residues.
(TIF)

S7 Fig *hrr25-2* leads to increased association of Hrr25 and Kti12 with Elongator. Elp1-HA was immunoprecipitated from extracts of the indicated *HRR25* and *hrr25-2* strains and immunoprecipitates were examined by Western blotting with anti-HA, anti-*myc* and anti-Hrr25 antibodies to detect immunoprecipitated Elp1 and co-immunoprecipitated Kti12 and Hrr25 respectively. The *ELP1* untagged control confirms that recovery of Kti12 and Hrr25 is dependent on Elp1.
(TIF)

S1 Table Summary of Zymocin phenotype monitored by Eclipse assay associated with different mutant *elp1* alleles. WAY034 (*elp1Δ*) and WAY031 (*elp1Δ sit4Δ*) were transformed with YCplac111, YCplac111-ELP1-6HA or with mutant derivatives of the latter plasmid and monitored by Eclipse assay for Zymocin sensitivity/resistance. R, Zymocin resistant; S, Zymocin sensitive; R/S, intermediate.
(PDF)

S2 Table Plasmids used or generated in this study.
(PDF)

S3 Table Yeast strains used or generated in this study.
(PDF)

## References

[1] Krogan NJ, Kim M, Ahn SH, Zhong G, Kobor MS, et al (2002) RNA polymerase II elongation factors of *Saccharomyces cerevisiae*: a targeted proteomics approach. Mol Cell Biol 22: 6979–6992. 12242279 10.1128/MCB.22.20.6979-6992.2002 PMC139818

[2] Otero G, Fellows J, Li Y, de Bizemont T, Dirac AM, et al (1999) Elongator, a multisubunit component of a novel RNA polymerase II holoenzyme for transcriptional elongation. Mol Cell 3: 109–118. 10024884 10.1016/s1097-2765(00)80179-3

[3] Chen YT, Hims MM, Shetty RS, Mull J, Liu L, et al (2009) Loss of mouse Ikbkap, a subunit of elongator, leads to transcriptional deficits and embryonic lethality that can be rescued by human IKBKAP. Mol Cell Biol 29: 736–744. 19015235 10.1128/MCB.01313-08 PMC2630687

[4] Simpson CL, Lemmens R, Miskiewicz K, Broom WJ, Hansen VK, et al (2009) Variants of the elongator protein 3 (*ELP3*) gene are associated with motor neuron degeneration. Hum Mol Genet 18: 472–481. 18996918 10.1093/hmg/ddn375 PMC2638803

[5] Strug LJ, Clarke T, Chiang T, Chien M, Baskurt Z, et al (2009) Centrotemporal sharp wave EEG trait in rolandic epilepsy maps to Elongator Protein Complex 4 (ELP4). Eur J Hum Genet 17: 1171–1181. 19172991 10.1038/ejhg.2008.267 PMC2729813

[6] Chen C, Tuck S, Bystrom AS (2009) Defects in tRNA modification associated with neurological and developmental dysfunctions in *Caenorhabditis elegans* elongator mutants. PLoS Genet 5: e1000561. 19593383 10.1371/journal.pgen.1000561 PMC2702823

[7] Solinger JA, Paolinelli R, Klöss H, Scorza FB, Marchesi S, et al (2010) The *Caenorhabditis elegans* Elongator complex regulates neuronal alpha-tubulin acetylation. PLoS Genet 6: e1000820. 20107598 10.1371/journal.pgen.1000820 PMC2809763

[8] Nelissen H, Fleury D, Bruno L, Robles P, De Veylder L, et al (2005) The *elongata* mutants identify a functional Elongator complex in plants with a role in cell proliferation during organ growth. Proc Natl Acad Sci USA 102: 7754–7759. 15894610 10.1073/pnas.0502600102 PMC1140448

[9] Nguyen L, Humbert S, Saudou F, Chariot A (2010) Elongator - an emerging role in neurological disorders. Trends Mol Med 16: 1–6. 20036197 10.1016/j.molmed.2009.11.002

[10] Torres AG, Batlle E, Ribas de Pouplana L (2014) Role of tRNA modifications in human diseases. Trends Mol Med 20: 306–314. 24581449 10.1016/j.molmed.2014.01.008

[11] Wittschieben BO, Otero G, de Bizemont T, Fellows J, Erdjument-Bromage H, et al (1999) A novel histone acetyltransferase is an integral subunit of elongating RNA polymerase II holoenzyme. Mol Cell 4: 123–128. 10445034 10.1016/s1097-2765(00)80194-x

[12] Winkler GS, Kristjuhan A, Erdjument-Bromage H, Tempst P, Svejstrup JQ (2002) Elongator is a histone H3 and H4 acetyltransferase important for normal histone acetylation levels *in vivo* . Proc Natl Acad Sci USA 99: 3517–3522. 11904415 10.1073/pnas.022042899 PMC122555

[13] Creppe C, Malinouskaya L, Volvert ML, Gillard M, Close P, et al (2009) Elongator controls the migration and differentiation of cortical neurons through acetylation of alpha-Tubulin. Cell 136: 551–564. 19185337 10.1016/j.cell.2008.11.043

[14] Miskiewicz K, Jose LE, Bento-Abreu A, Fislage M, Taes I, et al (2011) ELP3 controls active zone morphology by acetylating the ELKS family member Bruchpilot. Neuron 72: 776–788. 22153374 10.1016/j.neuron.2011.10.010

[15] Okada Y, Yamagata K, Hong K, Wakayama T, Zhang Y (2010) A role for the elongator complex in zygotic paternal genome demethylation. Nature 463: 554–558. 20054296 10.1038/nature08732 PMC2834414

[16] Frohloff F, Fichtner L, Jablonowski D, Breunig KD, Schaffrath R (2001) *Saccharomyces cerevisiae* Elongator mutations confer resistance to the *Kluyveromyces lactis* zymocin. EMBO J 20: 1993–2003. 11296232 10.1093/emboj/20.8.1993 PMC125238

[17] Li Q, Fazly AM, Zhou H, Huang S, Zhang Z, et al (2009) The Elongator complex interacts with PCNA and modulates transcriptional silencing and sensitivity to DNA damage agents. PLoS Genet 5: e1000684. 19834596 10.1371/journal.pgen.1000684 PMC2757915

[18] Jablonowski D, Schaffrath R (2007) Zymocin, a composite chitinase and tRNase killer toxin from yeast. Biochem Soc Trans 35: 1533–1537. 18031261 10.1042/BST0351533

[19] Rahl PB, Chen CZ, Collins RN (2005) Elp1p, the yeast homolog of the FD disease syndrome protein, negatively regulates exocytosis independently of transcriptional elongation. Mol Cell 17: 841–853. 15780940 10.1016/j.molcel.2005.02.018

[20] Huang B, Johansson MJ, Bystrom AS (2005) An early step in wobble uridine tRNA modification requires the Elongator complex. RNA 11: 424–436. 15769872 10.1261/rna.7247705 PMC1370732

[21] Mehlgarten C, Jablonowski D, Wrackmeyer U, Tschitschmann S, Sondermann D, et al (2010) Elongator function in tRNA wobble uridine modification is conserved between yeast and plants. Mol Microbiol 76: 1082–1094. 20398216 10.1111/j.1365-2958.2010.07163.x PMC2904499

[22] Lin F-J, Shen L, Jang C-W, Falnes PØ, Zhang Y (2013) Ikbkap/Elp1 Deficiency Causes Male Infertility by Disrupting Meiotic Progression. PLoS Genet 9: e1003516. 23717213 10.1371/journal.pgen.1003516 PMC3662645

[23] Lu J, Huang B, Esberg A, Johansson MJ, Bystrom AS (2005) The *Kluyveromyces lactis* gamma-toxin targets tRNA anticodons. RNA 11: 1648–1654. 16244131 10.1261/rna.2172105 PMC1370851

[24] Jablonowski D, Zink S, Mehlgarten C, Daum G, Schaffrath R (2006) tRNA^Glu^ wobble uridine methylation by Trm9 identifies Elongator's key role for zymocin-induced cell death in yeast. Mol Microbiol 59: 677–688. 16390459 10.1111/j.1365-2958.2005.04972.x

[25] Bjork GR, Huang B, Persson OP, Bystrom AS (2007) A conserved modified wobble nucleoside (mcm^5^s^2^U) in lysyl-tRNA is required for viability in yeast. RNA 13: 1245–1255. 17592039 10.1261/rna.558707 PMC1924908

[26] Agris PF (2008) Bringing order to translation: the contributions of transfer RNA anticodon-domain modifications. EMBO Rep 9: 629–635. 18552770 10.1038/embor.2008.104 PMC2475317

[27] Johansson MJ, Esberg A, Huang B, Bjork GR, Bystrom AS (2008) Eukaryotic wobble uridine modifications promote a functionally redundant decoding system. Mol Cell Biol 28: 3301–3312. 18332122 10.1128/MCB.01542-07 PMC2423140

[28] Esberg A, Huang B, Johansson MJ, Bystrom AS (2006) Elevated levels of two tRNA species bypass the requirement for elongator complex in transcription and exocytosis. Mol Cell 24: 139–148. 17018299 10.1016/j.molcel.2006.07.031

[29] Chen C, Huang B, Eliasson M, Ryden P, Bystrom AS (2011) Elongator Complex Influences Telomeric Gene Silencing and DNA Damage Response by Its Role in Wobble Uridine tRNA Modification. PLoS Genet 7: e1002258. 21912530 10.1371/journal.pgen.1002258 PMC3164696

[30] Rezgui VA, Tyagi K, Ranjan N, Konevega AL, Mittelstaet J, et al (2013) tRNA tKUUU, tQUUG, and tEUUC wobble position modifications fine-tune protein translation by promoting ribosome A-site binding. Proc Natl Acad Sci USA 110: 12289–12294. 23836657 10.1073/pnas.1300781110 PMC3725067

[31] Glatt S, Letoquart J, Faux C, Taylor NM, Seraphin B, et al (2012) The Elongator subcomplex Elp456 is a hexameric RecA-like ATPase. Nat Struct Mol Biol 19: 314–320. 22343726 10.1038/nsmb.2234

[32] Di Santo R, Bandau S, Stark MJR (2014) A conserved and essential basic region mediates tRNA binding to the Elp1 subunit of the *Saccharomyces cerevisiae* Elongator complex. Mol Microbiol 92: 1227–1242. 24750273 10.1111/mmi.12624 PMC4150532

[33] Paraskevopoulou C, Fairhurst SA, Lowe DJ, Brick P, Onesti S (2006) The Elongator subunit Elp3 contains a Fe_4_S_4_ cluster and binds *S*-adenosylmethionine. Mol Microbiol 59: 795–806. 16420352 10.1111/j.1365-2958.2005.04989.x

[34] Grove TL, Radle MI, Krebs C, Booker SJ (2011) Cfr and RlmN contain a single [4Fe-4S] cluster, which directs two distinct reactivities for *S*-adenosylmethionine: methyl transfer by SN2 displacement and radical generation. J Am Chem Soc 133: 19586–19589. 21916495 10.1021/ja207327v PMC3596424

[35] Selvadurai K, Wang P, Seimetz J, Huang RH (2014) Archaeal Elp3 catalyzes tRNA wobble uridine modification at C5 via a radical mechanism. Nat Chem Biol 10: 810–812. 25151136 10.1038/nchembio.1610 PMC4479141

[36] Jablonowski D, Butler AR, Fichtner L, Gardiner D, Schaffrath R, et al (2001) Sit4p protein phosphatase is required for sensitivity of *Saccharomyces cerevisiae* to *Kluyveromyces lactis* zymocin. Genetics 159: 1479–1489. 11779790 10.1093/genetics/159.4.1479 PMC1461913

[37] Mehlgarten C, Jablonowski D, Breunig KD, Stark MJR, Schaffrath R (2009) Elongator function depends on antagonistic regulation by casein kinase Hrr25 and protein phosphatase Sit4. Mol Microbiol 73: 869–881. 19656297 10.1111/j.1365-2958.2009.06811.x

[38] Mehlgarten C, Schaffrath R (2003) Mutant casein kinase I (Hrr25p/Kti14p) abrogates the G1 cell cycle arrest induced by *Kluyveromyces lactis* zymocin in budding yeast. Mol Genet Genomics 269: 188–196. 12756531 10.1007/s00438-003-0807-5

[39] Jablonowski D, Fichtner L, Stark MJR, Schaffrath R (2004) The yeast elongator histone acetylase requires Sit4-dependent dephosphorylation for toxin-target capacity. Mol Biol Cell 15: 1459–1469. 14718557 10.1091/mbc.E03-10-0750 PMC363168

[40] Soulard A, Cremonesi A, Moes S, Schutz F, Jeno P, et al (2010) The rapamycin-sensitive phosphoproteome reveals that TOR controls protein kinase A toward some but not all substrates. Mol Biol Cell 21: 3475–3486. 20702584 10.1091/mbc.E10-03-0182 PMC2947482

[41] Fukuchi S, Hosoda K, Homma K, Gojobori T, Nishikawa K (2011) Binary classification of protein molecules into intrinsically disordered and ordered segments. BMC Struc Biol 11: 29. 10.1186/1472-6807-11-29 PMC3199747 21693062

[42] Tyanova S, Cox J, Olsen J, Mann M, Frishman D (2013) Phosphorylation variation during the cell cycle scales with structural propensities of proteins. PLoS Comput Biol 9: e1002842. 23326221 10.1371/journal.pcbi.1002842 PMC3542066

[43] Butler AR, White JH, Folawiyo Y, Edlin A, Gardiner D, et al (1994) Two *Saccharomyces cerevisiae* genes which control sensitivity to G1 arrest induced by *Kluyveromyces lactis* toxin. Mol Cell Biol 14: 6306–6316. 8065362 10.1128/mcb.14.9.6306-6316.1994 PMC359157

[44] Huang B, Lu J, Bystrom AS (2008) A genome-wide screen identifies genes required for formation of the wobble nucleoside 5-methoxycarbonylmethyl-2-thiouridine in *Saccharomyces cerevisiae* . RNA 14: 2183–2194. 18755837 10.1261/rna.1184108 PMC2553728

[45] Fichtner L, Frohloff F, Burkner K, Larsen M, Breunig KD, et al (2002) Molecular analysis of *KTI12/TOT4*, a *Saccharomyces cerevisiae* gene required for *Kluyveromyces lactis* zymocin action. Mol Microbiol 43: 783–791. 11929532 10.1046/j.1365-2958.2002.02794.x

[46] Petrakis TG, Sogaard TM, Erdjument-Bromage H, Tempst P, Svejstrup JQ (2005) Physical and functional interaction between Elongator and the chromatin-associated Kti12 protein. J Biol Chem 280: 19454–19460. 15772087 10.1074/jbc.M413373200

[47] Zhu H, Klemic JF, Chang S, Bertone P, Casamayor A, et al (2000) Analysis of yeast protein kinases using protein chips. Nat Genet 26: 283–289. 11062466 10.1038/81576

[48] Gavin AC, Bosche M, Krause R, Grandi P, Marzioch M, et al (2002) Functional organization of the yeast proteome by systematic analysis of protein complexes. Nature 415: 141–147. 11805826 10.1038/415141a

[49] Schäfer T, Maco B, Petfalski E, Tollervey D, Bottcher B, et al (2006) Hrr25-dependent phosphorylation state regulates organization of the pre-40S subunit. Nature 441: 651–655. 16738661 10.1038/nature04840

[50] Wang X, Hoekstra MF, DeMaggio AJ, Dhillon N, Vancura A, et al (1996) Prenylated isoforms of yeast casein kinase I, including the novel Yck3p, suppress the *gcs1* blockage of cell proliferation from stationary phase. Mol Cell Biol 16: 5375–5385. 8816449 10.1128/mcb.16.10.5375 PMC231536

[51] Babu P, Deschenes RJ, Robinson LC (2004) Akr1p-dependent palmitoylation of Yck2p yeast casein kinase 1 is necessary and sufficient for plasma membrane targeting. J Biol Chem 279: 27138–27147. 15105419 10.1074/jbc.M403071200

[52] Robinson LC, Menold MM, Garrett S, Culbertson MR (1993) Casein kinase I-like protein kinases encoded by *YCK1* and *YCK2* are required for yeast morphogenesis. Mol Cell Biol 13: 2870–2881. 8474447 10.1128/mcb.13.5.2870-2881.1993 PMC359678

[53] Bodenmiller B, Wanka S, Kraft C, Urban J, Campbell D, et al (2010) Phosphoproteomic analysis reveals interconnected system-wide responses to perturbations of kinases and phosphatases in yeast. Sci Signal 3: rs4. 21177495 10.1126/scisignal.2001182 PMC3072779

[54] Kennelly PJ, Krebs EG (1991) Consensus sequences as substrate specificity determinants for protein kinases and protein phosphatases. J Biol Chem 266: 15555–15558. 1651913

[55] Corbett KD, Harrison SC (2012) Molecular architecture of the yeast monopolin complex. Cell Rep 1: 583–589. 22813733 10.1016/j.celrep.2012.05.012 PMC3494995

[56] Pfaffenwimmer T, Reiter W, Brach T, Nogellova V, Papinski D, et al (2014) Hrr25 kinase promotes selective autophagy by phosphorylating the cargo receptor Atg19. EMBO Rep 15: 862–870. 24968893 10.15252/embr.201438932 PMC4197043

[57] Ray P, Basu U, Ray A, Majumdar R, Deng H, et al (2008) The *Saccharomyces cerevisiae* 60S ribosome biogenesis factor Tif6p is regulated by Hrr25p-mediated phosphorylation. J Biol Chem 283: 9681–9691. 18256024 10.1074/jbc.M710294200 PMC2442299

[58] Bauer F, Matsuyama M, Candiracci J, Dieu M, Scheliga J, et al (2012) Translational control of cell division by elongator. Cell Rep 1: 424–433. 22768388 10.1016/j.celrep.2012.04.001 PMC3388810

[59] Zinshteyn B, Gilbert WV (2013) Loss of a Conserved tRNA Anticodon Modification Perturbs Cellular Signaling. PLoS Genet 9: e1003675. 23935536 10.1371/journal.pgen.1003675 PMC3731203

[60] Dedon PC, Begley TJ (2014) A system of RNA modifications and biased codon use controls cellular stress response at the level of translation. Chem Res Toxicol 27: 330–337. 24422464 10.1021/tx400438d PMC3997223

[61] Chan CT, Dyavaiah M, DeMott MS, Taghizadeh K, Dedon PC, et al (2010) A quantitative systems approach reveals dynamic control of tRNA modifications during cellular stress. PLoS Genet 6: e1001247. 21187895 10.1371/journal.pgen.1001247 PMC3002981

[62] Patil A, Dyavaiah M, Joseph F, Rooney JP, Chan CT, et al (2012) Increased tRNA modification and gene-specific codon usage regulate cell cycle progression during the DNA damage response. Cell Cycle 11: 3656–3665. 22935709 10.4161/cc.21919 PMC3478316

[63] Cyert MS (2003) Calcineurin signaling in *Saccharomyces cerevisiae*: how yeast go crazy in response to stress. Biochem Biophys Res Commun 311: 1143–1150. 14623300 10.1016/s0006-291x(03)01552-3

[64] Katis VL, Lipp JJ, Imre R, Bogdanova A, Okaz E, et al (2010) Rec8 phosphorylation by casein kinase 1 and Cdc7-Dbf4 kinase regulates cohesin cleavage by separase during meiosis. Devel Cell 18: 397–409. 20230747 10.1016/j.devcel.2010.01.014 PMC2994640

[65] Tanaka C, Tan LJ, Mochida K, Kirisako H, Koizumi M, et al (2014) Hrr25 triggers selective autophagy-related pathways by phosphorylating receptor proteins. J Cell Biol 207: 91–105. 25287303 10.1083/jcb.201402128 PMC4195827

[66] Ho Y, Mason S, Kobayashi R, Hoekstra M, Andrews B (1997) Role of the casein kinase I isoform, Hrr25, and the cell cycle-regulatory transcription factor, SBF, in the transcriptional response to DNA damage in *Saccharomyces cerevisiae* . Proc Natl Acad Sci USA 94: 581–586. 9012827 10.1073/pnas.94.2.581 PMC19556

[67] Begley U, Dyavaiah M, Patil A, Rooney JP, DiRenzo D, et al (2007) Trm9-Catalyzed tRNA Modifications link translation to the DNA damage response. Mol Cell 28: 860–870. 18082610 10.1016/j.molcel.2007.09.021 PMC2211415

[68] Amberg DC, Burke D, Strathern JN (2005) Methods in yeast genetics: a Cold Spring Harbor Laboratory course manual. New York: Cold Spring Harbor Laboratory Press.

[69] Sambrook J, Russell DW (2001) Molecular cloning: a laboratory manual: Cold Spring Harbor Laboratory Press.

[70] Van Driessche B, Tafforeau L, Hentges P, Carr AM, Vandenhaute J (2005) Additional vectors for PCR-based gene tagging in *Saccharomyces cerevisiae* and *Schizosaccharomyces pombe* using nourseothricin resistance. Yeast 22: 1061–1068. 16200506 10.1002/yea.1293

[71] Longtine MS, McKenzie A 3rd, Demarini DJ, Shah NG, Wach A, et al (1998) Additional modules for versatile and economical PCR-based gene deletion and modification in *Saccharomyces cerevisiae* . Yeast 14: 953–961. 9717241 10.1002/(SICI)1097-0061(199807)14:10<953::AID-YEA293>3.0.CO;2-U

[72] Knop M, Siegers K, Pereira G, Zachariae W, Winsor B, et al (1999) Epitope tagging of yeast genes using a PCR-based strategy: more tags and improved practical routines. Yeast 15: 963–972. 10407276 10.1002/(SICI)1097-0061(199907)15:10B<963::AID-YEA399>3.0.CO;2-W

[73] Séraphin B, Puig O, Bouveret E, Rutz B, Caspary F (2002) Tandem affinity purification to enhance interacting protein identification. In: Golemis E, editor. Protein-protein interactions: a molecular cloning manual. Cold Spring Harbor, New York: Cold Spring Harbor Laboratory Press. pp.313–328.

[74] Gietz RD, Sugino A (1988) New yeast-*Escherichia coli* shuttle vectors constructed with *in vitro* mutagenized yeast genes lacking six-base pair restriction sites. Gene 74: 527–534. 3073106 10.1016/0378-1119(88)90185-0

[75] Berben G, Dumont J, Gilliquet V, Bolle PA, Hilger F (1991) The YDp plasmids: a uniform set of vectors bearing versatile gene disruption cassettes for *Saccharomyces cerevisiae* . Yeast 7: 475–477. 1897313 10.1002/yea.320070506

[76] Kishida M, Tokunaga M, Katayose Y, Yajima H, Kawamura-Watabe A, et al (1996) Isolation and genetic characterization of pGKL killer-insensitive mutants (iki) from *Saccharomyces cerevisiae* . Biosci Biotechnol Biochem 60: 798–801. 8704309 10.1271/bbb.60.798

[77] Kellner S, Neumann J, Rosenkranz D, Lebedeva S, Ketting RF, et al (2014) Profiling of RNA modifications by multiplexed stable isotope labelling. Chem Commun 50: 3516–3518. 10.1039/c3cc49114e 24567952

[78] Bradford MM (1976) A rapid and sensitive method for the quantitation of microgram quantities of protein utilizing the principle of protein-dye binding. Anal Biochem 72: 248–254. 942051 10.1016/0003-2697(76)90527-3

[79] Zachariae W, Shin TH, Galova M, Obermaier B, Nasmyth K (1996) Identification of subunits of the anaphase-promoting complex of *Saccharomyces cerevisiae* . Science 274: 1201–1204. 8895471 10.1126/science.274.5290.1201

[80] Sutherland CM, Hawley SA, McCartney RR, Leech A, Stark MJR, et al (2003) Elm1p is one of three upstream kinases for the *Saccharomyces cerevisiae* SNF1 complex. Curr Biol 13: 1299–1305. 12906789 10.1016/s0960-9822(03)00459-7

[81] Hardie DG, Haystead TAJ, Salt IP, Davies SP (1999) Assay and purification of protein–serine/threonine kinases. In: Hardie DG, editor. Protein Phosphorylation: a Practical Approach. 2nd edition. Oxford: Oxford University Press. pp.201–220.

[82] Buchan DW, Minneci F, Nugent TC, Bryson K, Jones DT (2013) Scalable web services for the PSIPRED Protein Analysis Workbench. Nucleic Acids Res 41: W349–W357. 23748958 10.1093/nar/gkt381 PMC3692098

[83] Notredame C, Higgins DG, Heringa J (2000) T-Coffee: A novel method for fast and accurate multiple sequence alignment. J Mol Biol 302: 205–217. 10964570 10.1006/jmbi.2000.4042

[84] Fichtner L, Jablonowski D, Schierhorn A, Kitamoto HK, Stark MJR, et al (2003) Elongator's toxin-target (TOT) function is nuclear localization sequence dependent and suppressed by post-translational modification. Mol Microbiol 49: 1297–1307. 12940988 10.1046/j.1365-2958.2003.03632.x

