# Supplementary figures and images for: Phosphorylation of Elp1 by Hrr25 Is Required for Elongator-Dependent tRNA Modification in Yeast
Source: PLoS Genet. 2015 Jan 8;11(1):e1004931. doi: 10.1371/journal.pgen.1004931 (PMC4287497; doi:10.1371/journal.pgen.1004931)

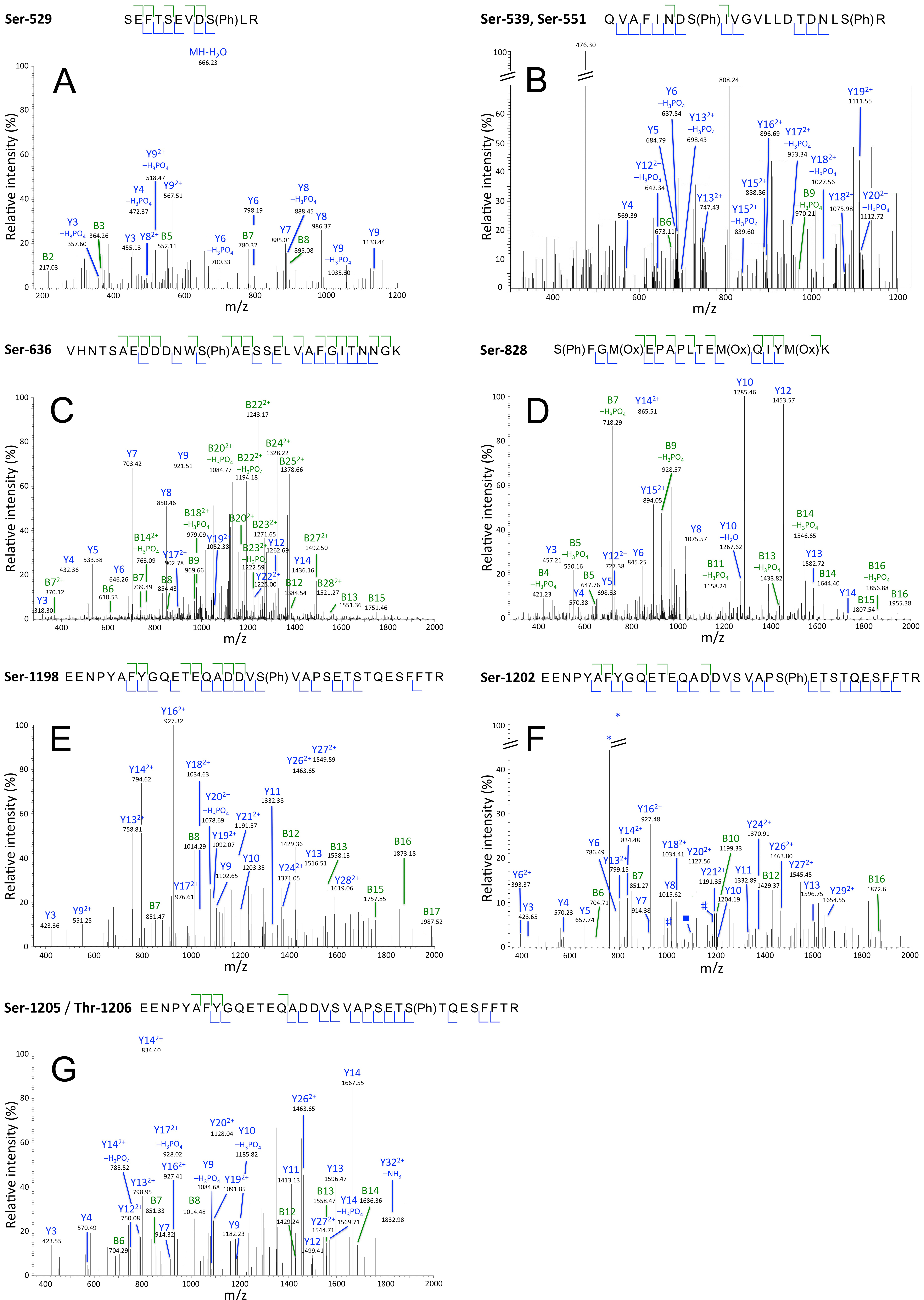

Supplement: S1 Fig — Mapping in vivo phosphorylation sites in yeast Elp1. Representative MS/MS spectra are shown in which diagnostic B (green) and Y (blue) ions are mapped onto the corresponding peptide sequence. S(Ph), phosphoserine; M(Ox), oxidized methionine. (A) Phosphorylation on Ser-529 within the Elp1 peptide 521-531, parent MH2+ ion m/z 675.29. (B) Dual phosphorylation on Ser-539 and Ser-551 within the Elp1 peptide 532-552, parent MH3+ ion m/z 817.41. (C) Phosphorylation on Ser-636 within the Elp1 peptide 624-652, parent MH3+ ion m/z 1063.46. (D) Phosphorylation on Ser-828 within the Elp1 peptide 828-844, parent MH2+ ion m/z 1051.44. (E) Phosphorylation on Ser-1198 within the Elp1 peptide 1180-1213, parent MH3+ ion m/z 1314.56. (F) Phosphorylation on Ser-1202 within the Elp1 peptide 1180-1213, parent MH2+ ion m/z 1314.56. This spectrum represents a mixture of monophosphorylated isoforms of the peptide: * denotes peaks consistent with Ser-1198 phosphorylation, # indicates peaks consistent with Ser-1205 phosphorylation and ▪ indicates an ion consistent with Thr-1206 phosphorylation. (G) Phosphorylation on Ser-1205 or Thr-1206 within the Elp1 peptide 1180-1213, parent MH2+ ion m/z 1314.56. (TIF) [file pgen.1004931.s001.tif]

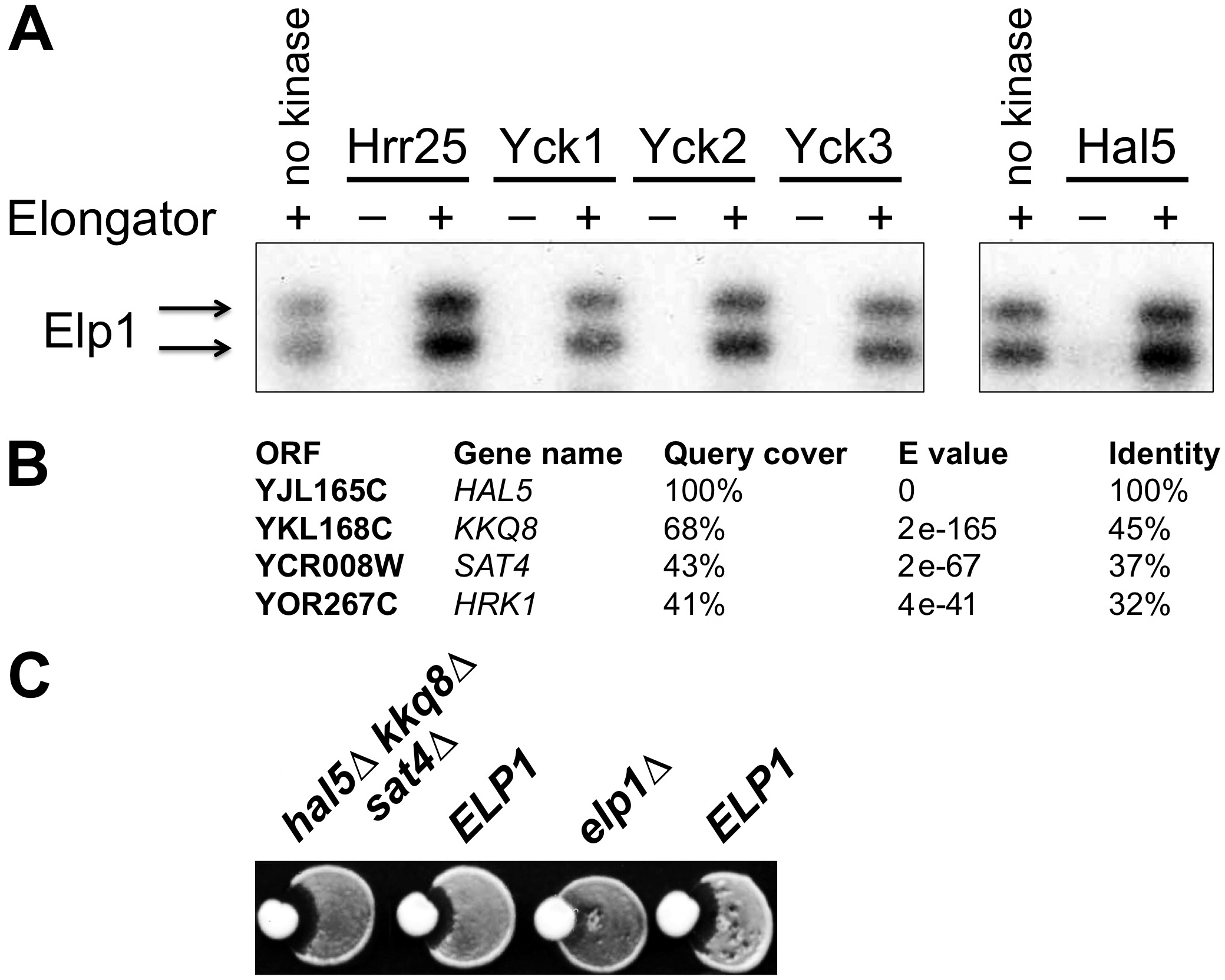

Supplement: S2 Fig — Identification of Elp1 kinases. (A) Phosphorylation of yeast Elongator by yeast protein kinases. Elongator was purified from yeast using TAP-tagged Elp3 and incorporation of 32P from [γ-32P]ATP in response to GST-kinase fusions identified from the GST-kinase library of Zhu et al. [47]. Background incorporation of 32P in the absence of added kinase is due to co-purification of Hrr25 with affinity-purified Elongator complex as shown in the main paper. Elp1 runs as a doublet, the faster-migrating component of which is truncated at the amino-terminus [84]. (B) Relatedness of Hal5 to Kkq8 and Sat4. A standard BLAST search (http://blast.ncbi.nlm.nih.gov/Blast.cgi) of the yeast proteome with Hal5 using the default parameters indicates that Kkq8 and Sat4 are its closest relatives within the yeast kinome. (C) Deletion of HAL5, either alone or in combination with KKQ8 and/or SAT4, fails to confer zymocin sensitivity by eclipse assay. (TIF) [file pgen.1004931.s002.tif]

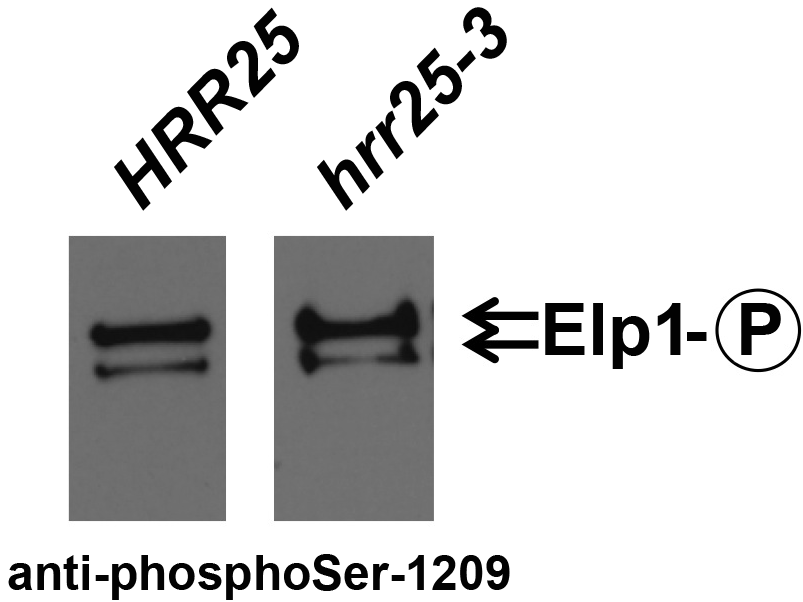

Supplement: S3 Fig — Elp1 shows similar levels of Ser-1209 phosphorylation in wild-type and hrr25-3 mutant strains. Elp1 phosphorylation in samples of extract containing equivalent amounts of protein was monitored by Western blot analysis using the phosphoSer-1209 phosphospecific antibody. (TIF) [file pgen.1004931.s003.tif]

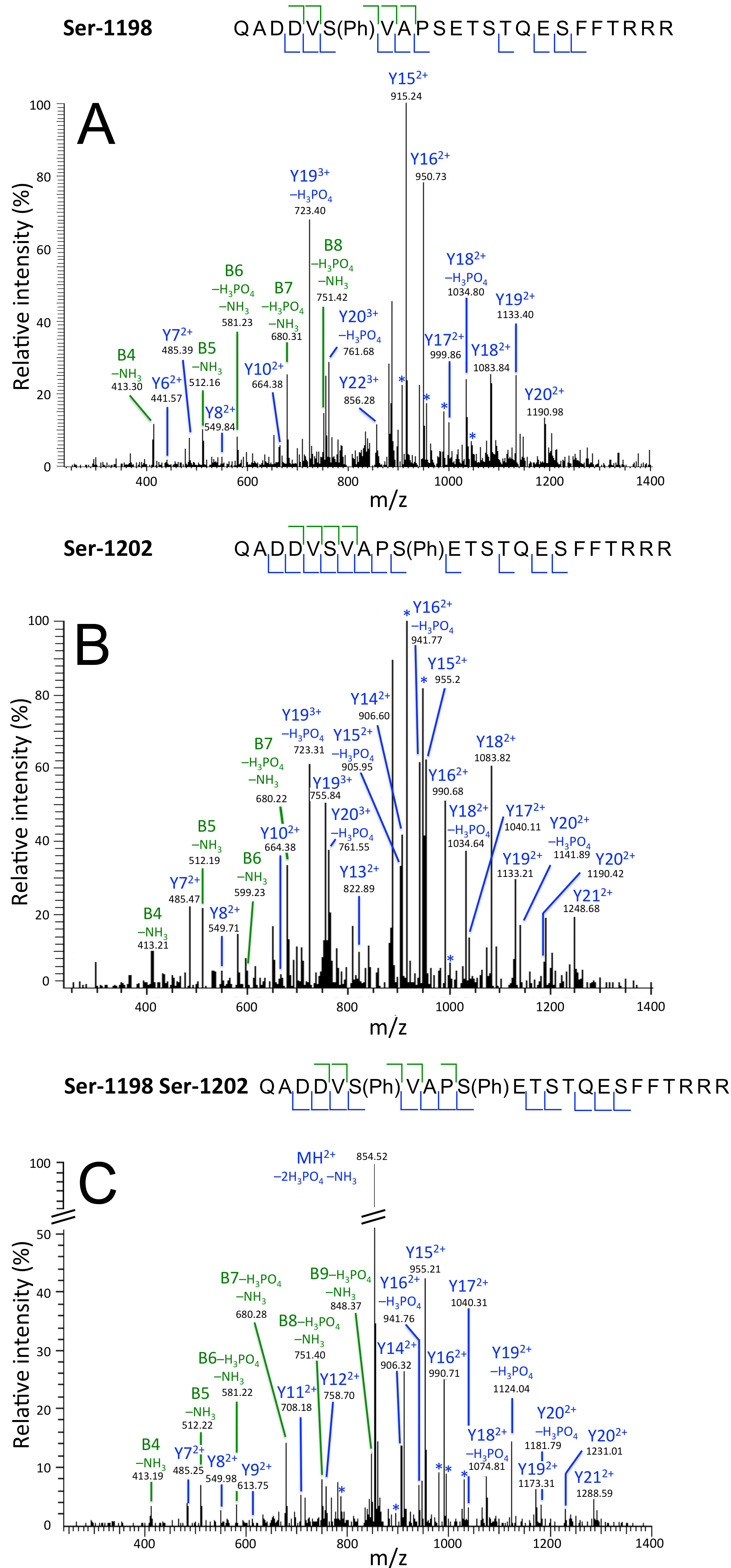

Supplement: S4 Fig — Mapping of phosphorylation sites following in vitro phosphorylation of a peptide corresponding to Elp1 residues 1193–1213. MS/MS spectra are shown in which diagnostic B (green) and Y (blue) ions are mapped onto the corresponding peptide sequence. S(Ph), phosphoserine. (A) Phosphorylation on Ser-1198, parent MH3+ ion m/z 889.08 corresponding to monophosphorylated peptide. * denotes peaks consistent with phosphorylation on Ser-1202, suggesting that this is a mixed spectrum. (B) Phosphorylation on Ser-1202, parent MH3+ ion m/z 889.08 corresponding to monophosphorylated peptide. * indicates peaks consistent with phosphorylation of Ser-1198, suggesting that this is a mixed spectrum. (C) Phosphorylation on Ser-1198 and Ser-1202, parent MH3+ ion m/z 925.73 corresponding to diphosphorylated peptide. * denotes peaks consistent with one phosphate on Ser-1202 and a second phosphate to the right, most likely on Ser-1205. (TIF) [file pgen.1004931.s004.tif]

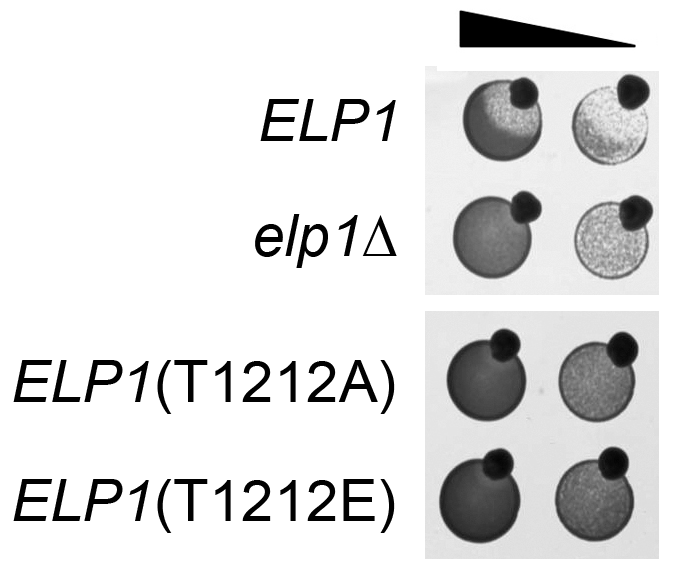

Supplement: S5 Fig — Neither alanine nor glutamate substitutions at Elp1 Thr-1212 support Elongator function. All strains were based on WAY034 transformed with YCplac111 (empty vector; elp1Δ), YCplac111-ELP1-6HA (ELP1 wild-type) or its Thr-1212 mutant derivatives as indicated. Zymocin sensitivity was measured by eclipse assay (see Fig. 1), testing samples of cells at either 1.0 or 0.1 OD600/ml following growth in SCD-Leu medium to select for the plasmids. (TIF) [file pgen.1004931.s005.tif]

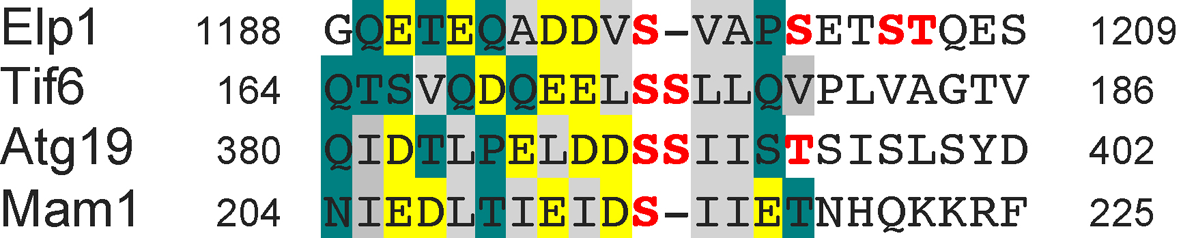

Supplement: S6 Fig — Alignment of Hrr25 phosphorylation sites. Alignment of the Elp1 Ser-1198 phosphorylation site with mapped Hrr25 phosphorylation sites in Tif6 [57], Atg19 [56] and Mam1 [55]. Sites known to be phosphorylated directly by Hrr25 are shown in red. Residues surrounding the mapped phosphorylation sites are highlighted in yellow (acidic), grey (small hydrophobic) or green (polar). In each case the phosphorylated residue is preceded by two acidic and one small hydrophobic residue and followed by two small hydrophobic residues. (TIF) [file pgen.1004931.s006.tif]

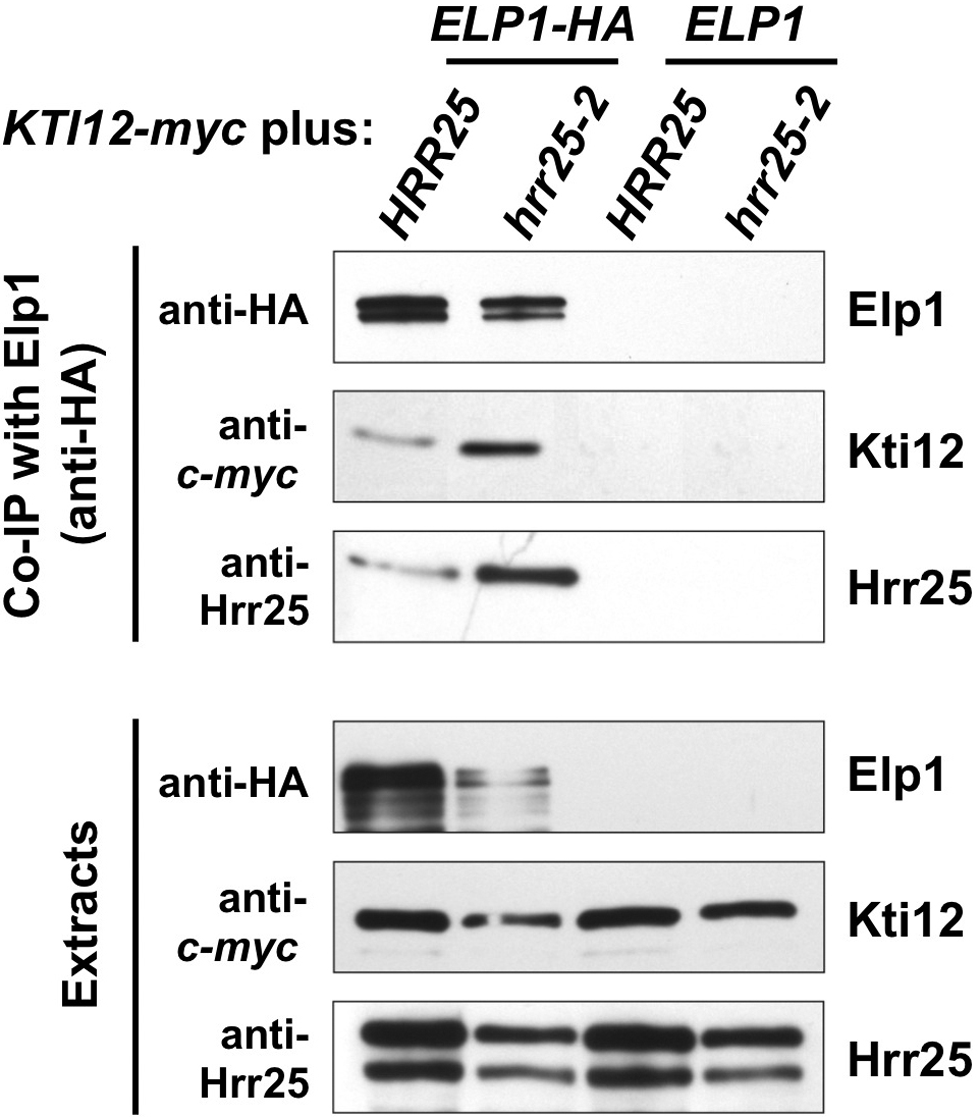

Supplement: S7 Fig — hrr25-2 leads to increased association of Hrr25 and Kti12 with Elongator. Elp1-HA was immunoprecipitated from extracts of the indicated HRR25 and hrr25-2 strains and immunoprecipitates were examined by Western blotting with anti-HA, anti-myc and anti-Hrr25 antibodies to detect immunoprecipitated Elp1 and co-immunoprecipitated Kti12 and Hrr25 respectively. The ELP1 untagged control confirms that recovery of Kti12 and Hrr25 is dependent on Elp1. (TIF) [file pgen.1004931.s007.tif]
