# Supplementary material for: Phosphorylation of Elp1 by Hrr25 Is Required for Elongator-Dependent tRNA Modification in Yeast
Source: PLoS Genet. 2015 Jan 8;11(1):e1004931. doi: 10.1371/journal.pgen.1004931 (PMC4287497; doi:10.1371/journal.pgen.1004931)
Supplement: S1 Table — Summary of Zymocin phenotype monitored by Eclipse assay associated with different mutant elp1 alleles. WAY034 (elp1Δ) and WAY031 (elp1Δ sit4Δ) were transformed with YCplac111, YCplac111-ELP1-6HA or with mutant derivatives of the latter plasmid and monitored by Eclipse assay for Zymocin sensitivity/resistance. R, Zymocin resistant; S, Zymocin sensitive; R/S, intermediate. (PDF) [file pgen.1004931.s008.pdf]

Table S1. Summary of zymocin phenotype monitored by eclipse assay associated with different mutant *elp1* alleles

**Plasmid**	***ELP1* mutations**	**zymocin phenotype**	
		elp1Δ	***elp1Δ sit4Δ***
YCplac111	Null (empty vector)	R	R
YCplac111-*ELP1*-6HA	None; *ELP1* wild-type	S	R
WAP008	S529A	S	
WAP007	S636A	S	
WAP828	S828A	S	
WAP059	T627A, S628A, S636A	S	R
WAP019	S1198A	S	R
WAP025	S1202A	S	R
WAP039 / DNA49	T1204A	S	
WAP038	S1205A	S	
DNA50	T1206A	S	
WAP068 / DNA6	S1209A	R	R
pRDS111	S529A, S539A, S551A	S	
WAP009	S529A, S636A, S828S, S1198A	S	R
WAP006	S529A, S636A, S828A, S1198A, S1202A	R	R
WAP027	S529A, S636A, S828A, S1198A, S1202A, S1205A, T1206A, S1209A	R	R
WAP010	S636A, S828S, S1198A	S	
WAP005	S1198A S1202A	R	R
WAP034	S1198A, T1206A, S1209A	R	R
WAP045	S1198A, S1205A, T1206A, S1209A	R	R
WAP046	S1202A, S1205A, T1206A, S1209A	R	R
pSS-AAA	T1204A, S1205A, T1206A	S	R
pAA-AAA	S1198A, S1202A, T1204A, S1205A, T1206A	R	R
WAP029	S1198A, S1202A, S1205A, T1206A, S1209A	R	R
WAP026	S1205A, T1206A, S1209A	R	
WAP011	S529E	S	R
WAP044	S828E	S	R
WAP013	S529E, S636E, S828E, S1198E, S1202E	S	R
WAP032	S1198E	S	R
WAP033	S1202E	S	R
WAP017 / DNA4	S1198E, S1202E	S	R
pSS-EDE	T1204E, S1205D, T1206E	S	
pEE-EDE	S1198E, S1202E, T1204E, S1205D, T1206E	S	
DNA7	S1209E	R/S	
DNA29	S1209D	S	R
WAP036	S1198E, S1205A, T1206A, S1209A	R	R
