# Supplementary material for: Phosphorylation of Elp1 by Hrr25 Is Required for Elongator-Dependent tRNA Modification in Yeast
Source: PLoS Genet. 2015 Jan 8;11(1):e1004931. doi: 10.1371/journal.pgen.1004931 (PMC4287497; doi:10.1371/journal.pgen.1004931)
Supplement: S2 Table — Plasmids used or generated in this study. (PDF) [file pgen.1004931.s009.pdf]

Table S2. Plasmids used or generated in this study

Name	Description	Source
pAE1	*CEN-ARS* p*GAL1*-zymocin γ expression plasmid	Ref. [1]
pCORE-UH	*KlURA3 Hyg^R* dual marker template	Ref. [2]
pFA6a-CTAP4-HIS3MX6	Template for C-terminal TAP tag addition with *HIS3MX* marker	Ashwin Bhat, unpublished
pFA6a-HIS3MX6	*HIS3MX* marker template	Ref. [3]
pGSKU	*KlURA3 KanMX6* dual marker template	Ref. [2]
pSB3	*ura3^{oc22} SUP4 natMX his3(495-620)::Msc*I*:: his3(211-304)*	Ref. [4]
pTrcHis-*HRR25*	*HRR25* in pTrcHis-TOPO®	Ref. [5]
YCplac111	*CEN4-ARS1 LEU2*	Ref. [6]
YCplac111-*ELP1*-6HA	YCplac111 containing the *ELP1* open reading frame with 632 bp of upstream sequence, fused to a 6HA tag and followed by the *K. lactis IPP1* transcription terminator	This study
YCplac111-*ELP1*-6HA Δ*Spe*I	YCplac111-*ELP1*-6HA with internal deletion between the two *Spe*I sites leaving 359 bp of upstream sequence before the *ELP1* ORF	This study
pRDS111	YCplac111-*ELP1*-6HAΔ*Spe*I but *elp1*(S529A, S539A, S551A)	This study
WAP006	YCplac111-*ELP1*-6HA but *elp1*(S529A, S636A, S828A, S1198A, S1202A)	This study
WAP007	YCplac111-*ELP1*-6HA but *elp1*(S636A)	This study
WAP008	YCplac111-*ELP1*-6HA but *elp1*(S529A)	This study
WAP009	YCplac111-*ELP1*-6HA but *elp1*(S529A, S636A, S828A, S1198A)	This study
WAP019	YCplac111-*ELP1*-6HA but *elp1*(S1198A)	This study
WAP025	YCplac111-*ELP1*-6HA but *elp1*(S1202A)	This study
WAP038	YCplac111-*ELP1*-6HA but *elp1*(S1205A)	This study
WAP039	YCplac111-*ELP1*-6HA but *elp1*(T1204A)	This study
WAP1206	YCplac111-*ELP1*-6HA but *elp1*(T1206A)	This study
WAP828	YCplac111-*ELP1*-6HA but *elp1*(S828A)	This study
pDNA0	YCplac111-*ELP1*-6HA but *elp1*(S1198Stop)	This study
pDNA1	YCplac111-*ELP1*-6HA but *elp1*(S1198A, S1202A)	This study
pDNA4	YCplac111-*ELP1*-6HA but *elp1*(S1198E, S1202E)	This study
pDNA5	YCplac111-*ELP1*-6HA but *elp1*(T1204A, S1205A, T1206A)	This study
pDNA6	YCplac111-*ELP1*-6HA but *elp1*(S1209A)	This study
pDNA7	YCplac111-*ELP1*-6HA but *elp1*(S1209E)	This study
pDNA13	YCplac111-*ELP1*-6HA but *elp1*(T1212A)	This study
pDNA29	YCplac111-*ELP1*-6HA but *elp1*(S1209D)	This study
pDNA35	YCplac111-*ELP1*-6HA but *elp1*(S1212E)	This study
pAA-AAA	YCplac111-*ELP1*-6HA but *elp1*(S1198A, S1202A, T1204A, S1205A, T1206A)	This study
pAA-EDE	YCplac111-*ELP1*-6HA but *elp1*(S1198A, S1202A, T1204E, S1205D, T1206E)	This study

pEE-EDE	YCplac111-*ELP1*-6HA but *elp1*(S1198E, S1202E, T1204E, S1205D, T1206E)	This study
pSS-EDE	YCplac111-*ELP1*-6HA but *elp1*(T1204E, S1205D, T1206E)	This study

1. Butler AR, White JH, Folawiyo Y, Edlin A, Gardiner D, et al. (1994) Two *Saccharomyces cerevisiae* genes which control sensitivity to G1 arrest induced by *Kluyveromyces lactis* toxin. Mol Cell Biol 14: 6306-6316.
2. Storici F, Resnick MA (2006) The *delitto perfetto* approach to *in vivo* site-directed mutagenesis and chromosome rearrangements with synthetic oligonucleotides in yeast. Methods Enzymol 409: 329-345.
3. Longtine MS, McKenzie A, 3rd, Demarini DJ, Shah NG, Wach A, et al. (1998) Additional modules for versatile and economical PCR-based gene deletion and modification in *Saccharomyces cerevisiae*. Yeast 14: 953-961.
4. Di Santo R, Bandau S, Stark MJR (2014) A conserved and essential basic region mediates tRNA binding to the Elp1 subunit of the *Saccharomyces cerevisiae* Elongator complex. Mol Microbiol 92: 1227-1242.
5. Mehlgarten C, Jablonowski D, Breunig KD, Stark MJ, Schaffrath R (2009) Elongator function depends on antagonistic regulation by casein kinase Hrr25 and protein phosphatase Sit4. Mol Microbiol 73: 869-881.
6. Gietz RD, Sugino A (1988) New yeast-*Escherichia coli* shuttle vectors constructed with *in vitro* mutagenized yeast genes lacking six-base pair restriction sites. Gene 74: 527-534.
