# Supplementary material for: Phosphorylation of Elp1 by Hrr25 Is Required for Elongator-Dependent tRNA Modification in Yeast
Source: PLoS Genet. 2015 Jan 8;11(1):e1004931. doi: 10.1371/journal.pgen.1004931 (PMC4287497; doi:10.1371/journal.pgen.1004931)
Supplement: S3 Table — Yeast strains used or generated in this study. (PDF) [file pgen.1004931.s010.pdf]

Table S3. Yeast strains used or generated in this study

Name	Genotype	Source
K. lactis		
NCYC1368	*MAT***a** prototroph pGLK2$^+$ pGLK1$^+$	NCYC [1]
S. cerevisiae		
ANY21	*MAT***a** *ura3-52 leu2-3,112 his3 his4 trp1-289*	Ref. [2]
AMY11-2C	ANY21 *hrr25-2* [T176I]	Ref. [2]
ARB97	*MATα leu2-3,112 his3-11,15 hrr25-3 (kti14-1) can1*	Ref. [3]
ARB97 *elp1Δ*	ARB97 *elp1Δ::HIS3MX6*	This study
BY4741	*MAT***a** *his3Δ1 leu2Δ0 met15Δ0 ura3Δ0*	Ref. [4]
BY4742	*MATα his3Δ1 leu2Δ0 lys2Δ0 ura3Δ0*	Ref. [4]
DJY500	ANY21 *ELP1-HA*$_6$*::KlTRP1*	This study
DJY501	ANY21 *ELP1-HA*$_6$*::KlTRP1 KTI12-myc*$_3$*::SpHis5*	This study; from DJY500
DJY502	ANY21 *hrr25-2 ELP1-HA*$_6$*::KlTRP1*	This study; from AMY11-2C
DJY503	ANY21 *hrr25-2 ELP1-HA*$_6$*::KlTRP1 KTI12-myc*$_3$*::SpHis5*	This study; from DJY502
DJY504	FY1679-08A *ELP2-myc*$_3$*::SpHis5 ELP3-HA*$_6$*::KlTRP1 elp1Δ::KanMX6*	This study; from FFY2/3-dt
DJY505	FY1679-08A *ELP2-myc*$_3$*::SpHis5 ELP5-HA*$_6$*::KlTRP1 elp1Δ::KanMX6*	This study; from FFY2/5-dt
DJY506	FY1679-08A *ELP2-myc*$_3$*::SpHis5 KTI12-(HA)*$_6$*::KlTRP1 elp1Δ::KanMX6*	This study; from FFY2/4-dt
FFY2/3-dt	FY1679-08A *ELP2-myc*$_3$*::SpHis5 ELP3-HA*$_6$*::KlTRP1*	Ref. [5]
FFY2/4-dt	FY1679-08A *ELP2-myc*$_3$*::SpHis5 KTI12-HA*$_6$*::KlTRP1*	Ref. [5]
FFY2/5-dt	FY1679-08A *ELP2-myc*$_3$*::SpHis5 ELP5-HA*$_6$*::KlTRP1*	Ref. [5]
FY1679-08A	*MAT***a** *ura3-52 leu2Δ1 trp1Δ63 his3Δ200*	Ref. [6]
RL-343-F0	BY4741 *hrr25::KanMX6* [pJU1163 (pRS416 + *HRR25*)]	Ref. [7]
RL-343-F1	BY4741 *hrr25::KanMX6* [pJU1197 (pRS416 + *hrr25 I82G*)]	Ref. [7]
SBY138	BY4741 *his3Δ1::*pSB3 (*NatMX SUP4 ura3*^{oc22}) *elp1Δ::KanMX6*	This study; from WAY034
WAY008	BY4741 *ELP3-TAP::HIS3MX*	Open Biosystems YSC1178-202233681
WAY009	BY4741 *ELP1-TAP::HIS3MX*	Open Biosystems YSC1178-202232517
WAY010	BY4741 *sit4Δ::LEU2 ELP1-TAP::HIS3MX*	This study; from WAY009
WAY011	BY4741 *kti12Δ::LEU2 ELP1-TAP::HIS3MX*	This study; from WAY009
WAY031	BY4742 *sit4Δ::kanMX6 elp1Δ::pGSUK*	This study
WAY034	BY4741 *elp1Δ::KanMX6*	This study
WAY037	BY4741 *elp1Δ::pCORE-UH (KlURA3 Hyg*^{r}*) ELP3-TAP::HIS3MX*	This study; from WAY008
WAY-H-P1T	BY4741 *hrr25::KanMX6 ELP1-TAP::HIS3MX* [pJU1163 (pRS416 + *HRR25*)]	This study; from RL-343-F0

WAY-Has-P1T	BY4741 *hrr25::KanMX6 ELP1-TAP::HIS3MX* [pJU1197 (pRS416 + *hrr25 I82G*)]	This study; from RL-343-F1
YRDS461	BY4741 *ELP1*-GFP-*HIS3MX KTI12* HA_3-*KanMX6 NIC96*-mCherry$_4$*::NatRMX*	Ref. [8]
YRDS467	BY4741 *elp1-KR9A-GFP-HIS3MX KTI12* HA_3-*KanMX6 NIC96*-mCherry$_4$*::NatRMX*	Ref. [8]
YRDS529	BY4741 *elp1(S1209A)*-GFP-*HIS3MX KTI12* HA_3-*KanMX6 NIC96*-mCherry$_4$*::NatRMX*	This study

1. National Collection of Yeast Cultures [http://www.ncyc.co.uk].
2. Murakami A, Kimura K, Nakano A (1999) The inactive form of a yeast casein kinase I suppresses the secretory defect of the *sec12* mutant. Implication of negative regulation by the Hrr25 kinase in the vesicle budding from the endoplasmic reticulum. J Biol Chem 274: 3804-3810.
3. Butler AR, White JH, Folawiyo Y, Edlin A, Gardiner D, et al. (1994) Two *Saccharomyces cerevisiae* genes which control sensitivity to G1 arrest induced by *Kluyveromyces lactis* toxin. Mol Cell Biol 14: 6306-6316.
4. Brachmann CB, Davies A, Cost GJ, Caputo E, Li J, et al. (1998) Designer deletion strains derived from *Saccharomyces cerevisiae* S288C: a useful set of strains and plasmids for PCR-mediated gene disruption and other applications. Yeast 14: 115-132.
5. Fichtner L, Frohloff F, Burkner K, Larsen M, Breunig KD, et al. (2002) Molecular analysis of *KTI12/TOT4*, a *Saccharomyces cerevisiae* gene required for *Kluyveromyces lactis* zymocin action. Mol Microbiol 43: 783-791.
6. Euroscarf [http://web.uni-frankfurt.de/fb15/mikro/euroscarf/].
7. Bodenmiller B, Wanka S, Kraft C, Urban J, Campbell D, et al. (2010) Phosphoproteomic analysis reveals interconnected system-wide responses to perturbations of kinases and phosphatases in yeast. Sci Signal 3: rs4.
8. Di Santo R, Bandau S, Stark MJR (2014) A conserved and essential basic region mediates tRNA binding to the Elp1 subunit of the *Saccharomyces cerevisiae* Elongator complex. Mol Microbiol 92: 1227-1242.
